# Calcium Orthophosphate Cements and Concretes

**DOI:** 10.3390/ma2010221

**Published:** 2009-03-19

**Authors:** Sergey V. Dorozhkin

**Affiliations:** Kudrinskaja sq. 1-155, Moscow 123242, Russia; E-Mail: sedorozhkin@yandex.ru; Tel. +7-499-255-4460

**Keywords:** Calcium orthophosphate, cement, concrete, self-setting, hydraulic, injectable, bone grafting, dental, reinforced, calcium deficient hydroxyapatite, brushite, bioceramics, biomaterials, osteoconductivity, bioresorbability, materials science

## Abstract

In early 1980s, researchers discovered self-setting calcium orthophosphate cements, which are a bioactive and biodegradable grafting material in the form of a powder and a liquid. Both phases form after mixing a viscous paste that after being implanted, sets and hardens within the body as either a non-stoichiometric calcium deficient hydroxyapatite (CDHA) or brushite, sometimes blended with unreacted particles and other phases. As both CDHA and brushite are remarkably biocompartible and bioresorbable (therefore, *in vivo* they can be replaced with newly forming bone), calcium orthophosphate cements represent a good correction technique for non-weight-bearing bone fractures or defects and appear to be very promising materials for bone grafting applications. Besides, these cements possess an excellent osteoconductivity, molding capabilities and easy manipulation. Furthermore, reinforced cement formulations are available, which in a certain sense might be described as calcium orthophosphate concretes. The concepts established by calcium orthophosphate cement pioneers in the early 1980s were used as a platform to initiate a new generation of bone substitute materials for commercialization. Since then, advances have been made in the composition, performance and manufacturing; several beneficial formulations have already been introduced as a result. Many other compositions are in experimental stages. In this review, an insight into calcium orthophosphate cements and concretes, as excellent biomaterials suitable for both dental and bone grafting application, has been provided.

## 1. Introduction

Calcium orthophosphates have been studied as bone repair materials for the last 80 years. The first *in vivo* use of calcium orthophosphates was performed in 1920, when researchers implanted tricalcium phosphate (TCP) into animals to test its efficacy as a bone substitute [1]. In the following years, some other calcium orthophosphates were tested on animals to investigate their effect on the healing of nonunions [2]. However, it was 1951, when for the first time hydroxyapatite (HA) was implanted in rats and guinea pigs [3]. Those attempts might be characterized as the initial medical trials with the first generation of bone substituting biomaterials. However, it was already the 1970s, when other calcium orthophosphates were synthesized, characterized, investigated and tried in medicine [4,5,6,7,8,9,10]. The list of known calcium orthophosphates, including their standard abbreviations and the major properties, is shown in Table 1 [11].

The possibility of obtainng a monolithic calcium orthophosphate ceramics at ambient or body temperature via a cementation reaction was put forward by the scientists at the American Dental Association LeGeros *et al*. [12] and Brown and Chow [13,14,15,16] in the early 1980s. However, there is an opinion [17] that the self-setting calcium orthophosphate cements for orthopedic and dental restorative applications were first described in the early 1970s by Driskell *et al*. [18]. More to the point, some researchers worked with similar reactions even earlier. For example, Kingery looked at formulations based on CaO and H_3_PO_4_ in 1950 [19]. Currently this type of materials is known as *calcium phosphate cements* (commonly referred to as CPC), and, due to their suitability for repair, augmentation and regeneration of bones, they might be named as calcium phosphate *bone* cements (occasionally referred to as CPBC) [20]. In order to stress the fact that these cements consist either entirely or essentially of calcium *ortho*phosphates, this review is limited to consideration of calcium orthophosphate cements only. Due to a good bioresorbability, calcium orthophosphate cements belong to the second generation of bone substituting biomaterials [21]. These cements are blends of amorphous and/or crystalline calcium orthophosphate powder(s) with an aqueous solution, which might be distilled water, phosphate-buffered saline (PBS), ~ 0.25 M aqueous solution of sodium orthophosphate, ortho-phosphoric acid, ~ 0.5 M aqueous solution of citric acid [22] or even revised simulated body fluid (rSBF) [23].

After the powder(s) and the solution are mixed together, a viscous and moldable paste is formed that sets to a firm mass within a few minutes. When the paste becomes sufficiently stiff, it can be placed into a defect as a substitute for the damaged part of bone, where it hardens *in situ* within the operating theatre. The proportion of solid to liquid or the powder-to-liquid (P/L) ratio is a very important characteristic because it determines both bioresorbability and rheological properties. As the paste is set and hardened at room or body temperature, direct application in healing of bone defects became a new and innovative treatment option by the end of the XX-th century. Moreover, calcium orthophosphate cements can be injected directly into the fractures and bone defects, where they intimately adapt to the bone cavity regardless its shape. More to the point, they were found to promote development of osteoconductive pathways, possess sufficient compressive strengths, be noncytotoxic, create chemical bonds to the host bones, restore contour and have both the chemical composition and X-ray diffraction patterns similar to those of bone [24]. Finally, yet importantly, they are osteotransductive, *i.e*., after implantation, calcium orthophosphate cements are replaced by a new bone tissue [25,26,27].

The aim of biomimetic bone cements is to disturb bone functions and properties as little as possible and, until a new bone has been grown, to behave temporarily in a manner similar to that of bone. From a biological point of view, this term defines cements that can reproduce the composition, structure, morphology and crystallinity of bone crystals [28,29]. Therefore, the discovery of self-setting calcium orthophosphate cements was a significant step forward in the field of bioceramics for bone regeneration, since it established good prospects for minimally invasive surgical techniques that were less aggressive than the classical surgical methods [30]. The cements provide the surgeons with a unique ability of manufacturing, shaping and implanting the bioactive bone substitute material on a patient-specific base in real time in the surgery room. Implanted bone tissues also take benefits from initial setting characteristics of the cements that give, in an acceptable clinical time, a suitable mechanical strength for a shorter tissue functional recovery. The major advantages of the cements include a fast setting time, excellent moldability, outstanding biocompatibility and easy manipulation; therefore, the cements are more versatile in handling characteristics than prefabricated calcium orthophosphate granules or blocks. Besides, like any other bioceramics, calcium orthophosphate cements provide the opportunity for bone grafting using alloplastic materials, which are unlimited in quantity and provide no risk of infectious diseases [31,32,33].

From the point of view that calcium orthophosphate cements are intended for use as biomaterials for parenteral application, in their chemical composition one might employ any ionic compounds of oligoelements occurring naturally in a human body. The list of possible additives includes (but is not limited to) the following cations: Na^+^, K^+^, Mg^2+^, Ca^2+^, Sr^2+^, H^+^ and anions: PO_4_^3−^, HPO_4_^2−^, H_2_PO_4_^−^, P_2_O_7_^4−^, CO_3_^2−^, HCO_3_^−^, SO_4_^2−^, HSO_4_^−^, Cl^−^, OH^−^, F^−^, SiO_4_^4−^ [25]. Therefore, mixed-type cements consisting of calcium orthophosphates and other calcium salts (*e.g.,* gypsum [34,38,39], calcium sulfate hemihydrate [40], calcium pyrophosphate [41,42,43], calcium polyphosphates [44], calcium carbonate [29,45,46,47], calcium oxide [48,49,50,51,52,53], calcium hydroxide [54,55,56], calcium aluminate [57,58], calcium silicate [59,60,61,62], *etc*.), strontium orthophosphate [63,64], magnesium orthophosphate [65,66,67], barium sulfate [68], as well as cements made of various ion substituted calcium orthophosphates (*e.g.,* Ca_2_KNa(PO_4_)_2_, NaCaPO_4_, Na_3_Ca_6_(PO_4_)_5_, magnesium substituted CDHA, strontium substituted CDHA, *etc*.) [69,70,71,72,73,74,75,76,77] are available. Moreover, calcium orthophosphate cements might be prepared in the reaction-setting mixture of Ca(OH)_2_–KH_2_PO_4_ system [78], as well as by treatment of calcium carbonates with orthophosphate solutions [79]. Calcium orthophosphate cements possessing magnetic properties due to incorporation of iron oxides have been developed as well [80,81]. However, with a few important exceptions, such formulations have not been considered in this review, whose purpose it is to review the chemistry, physical and mechanical properties of calcium orthophosphate cements with specific reference to their biomedical applications in dentistry and surgery.

**Table 1:** Existing calcium orthophosphates and their major properties [11].

Ca/P ionic ratio	Compound and its abbreviation	Chemical formula	Solubility at 25 °C, – log(K_s_)	Solubility at 25 °C, g/L	Stability in aqueous solutions at 25 °C (pH range)
0.5	Monocalcium phosphate monohydrate (MCPM)	Ca(H_2_PO_4_)_2_·H_2_O	1.14	~ 18	0.0 – 2.0
0.5	Monocalcium phosphate anhydrous (MCPA)	Ca(H_2_PO_4_)_2_	1.14	~ 17	^[c]^
1.0	Dicalcium phosphate dihydrate (DCPD), mineral brushite	CaHPO_4_·2H_2_O	6.59	~ 0.088	2.0 – 6.0
1.0	Dicalcium phosphate anhydrous (DCPA), mineral monetite	CaHPO_4_	6.90	~ 0.048	^[c]^
1.33	Octacalcium phosphate (OCP)	Ca_8_(HPO_4_)_2_(PO_4_)_4_·5H_2_O	96.6	~ 0.0081	5.5 – 7.0
1.5	α-Tricalcium phosphate (α-TCP)	α-Ca_3_(PO_4_)_2_	25.5	~ 0.0025	^[a]^
1.5	β-Tricalcium phosphate (β-TCP)	β-Ca_3_(PO_4_)_2_	28.9	~ 0.0005	^[a]^
1.2 – 2.2	Amorphous calcium phosphate (ACP)	Ca_x_H_y_(PO_4_)_z_·nH_2_O, n = 3 – 4.5; 15 – 20% H_2_O	^[b]^	^[b]^	~ 5 – 12 ^[d]^
1.5 – 1.67	Calcium-deficient hydroxyapatite (CDHA)^[e]^	Ca_10-*x*_(HPO_4_)*_x_*(PO_4_)_6-*x*_(OH)_2-*x*_^[f]^ (0 < *x* < 1)	~ 85.1	~ 0.0094	6.5 – 9.5
1.67	Hydroxyapatite (HA)	Ca_10_(PO_4_)_6_(OH)_2_	116.8	~ 0.0003	9.5 – 12
1.67	Fluorapatite (FA)	Ca_10_(PO_4_)_6_F_2_	120.0	~ 0.0002	7 – 12
2.0	Tetracalcium phosphate (TTCP), mineral hilgenstockite	Ca_4_(PO_4_)_2_O	38 - 44	~ 0.0007	^[a]^

^[a]^ These compounds cannot be precipitated from aqueous solutions. ^[b]^ Cannot be measured precisely. However, the following values were found: 25.7 ± 0.1 (pH = 7.40), 29.9 ± 0.1 (pH = 6.00), 32.7 ± 0.1 (pH = 5.28). ^[c]^ Stable at temperatures above 100 °C. ^[d]^ Always metastable. ^[e]^ Occasionally, CDHA is named as precipitated HA. ^[f]^ In the case *x* = 1 (the boundary condition with Ca/P = 1.5), the chemical formula of CDHA looks as follows: Ca_9_(HPO_4_)(PO_4_)_5_(OH).

## 2. Calcium Orthophosphate Cements

### 2.1. General Information and Brief History

According to the free encyclopedia Wikipedia: “In the most general sense of the word, *cement* is a binder, a substance that sets and hardens independently and can bind other materials together. The name “cement” goes back to the Romans who used the term “*opus caementitium*” to describe masonry which resembled concrete and was made from crushed rock with burnt lime as binder. Volcanic ash and pulverized brick additives, which were added to the burnt lime to obtain a hydraulic binder, were later referred to as cementum, cimentum, cäment and cement” [82]. Thus, *calcium orthophosphate cement* appears to be a generic term to describe chemical formulations in the ternary system Ca(OH)_2_ – H_3_PO_4_ – H_2_O which can experience a transformation from a liquid or pasty state to a solid state and in which the end-product of the chemical reactions is a calcium orthophosphate.

The first calcium orthophosphate cement formulation consisted of an equimolar mixture of TTCP and dicalcium phosphate (DCPA or DCPD) [83] which is mixed with water at a P/L ratio of 4:1; the paste hardened in about 30 min and formed CDHA [14,15]. This highly viscous, non-injectable paste can be molded and is therefore used mainly as a contouring material in craniofacial surgery. In the 1990s, it was established that there were about 15 different binary combinations of calcium orthophosphates, which gave pastes upon mixing with water or aqueous solutions, so that the pastes set at room or body temperature into a solid cement. The list of these combinations is available in literature [86,87,88]. From these basic systems, secondary formulations containing additional or even non-reactive compounds but still setting like cements could be derived [25,50,86,89,90,91,92,93,94,95,96,97,98,99,100,101,102]. According to the classical solubility data, depending upon the pH value of a cement paste, after setting all calcium orthophosphate cements can only form two major end-products: a precipitated poorly crystalline HA or CDHA [103] at pH > 4.2 and DCPD (also called “brushite” [104]) at pH < 4.2 [105]. However, in the real cement formulations this pH-border is shifted to a higher value of pH. Namely, DCPD might be formed at pH up to ~ 6, while CDHA normally is not formed at pHs below 6.5 – 7 (Table 1). The results of the only study on an ACP cement [100] demonstrated that this end-product was rapidly converted into CDHA. Besides, there is one paper devoted to an OCP cement [106]; however, contrary to reports in the early 1990s, none of the calcium orthophosphate cements synthesized afterwards was of OCP or ACP types. Therefore, all existing formulations of calcium orthophosphate cements have been divided into two major groups: apatite cements and brushite cements [107]. The final hardened product of the cements is of the paramount importance because it determines the solubility and, therefore, *in vivo* bioresorbability. Since the chemical composition of mammalian bones is similar to an ion-substituted CDHA, apatite cements have been more extensively investigated. However, many research papers on brushite cements have been published as well.

### 2.2. Composition and Crystallization

All calcium orthophosphate cements are made of an aqueous solution and fine powders of one or several calcium orthophosphate(s). Here, dissolution of the initial calcium orthophosphates (quickly or slowly, depending on the chemical composition and solution pH) and mass transport appear to be the primary functions of an aqueous environment, in which the dissolved reactants form a supersaturated (very far away from the equilibrium) microenvironment with regard to precipitation of the final products [109,110]. The relative stability and solubility of various calcium orthophosphates is the major driving force for the setting reactions that occur in these cements. Therefore, mixing of a dry powder with an aqueous solution induces various chemical transformations, where crystals of the initial calcium orthophosphate(s) rapidly dissolve(s) and precipitate(s) into crystals of CDHA or DCPD, with possible formation of intermediate precursor phases (*e.g.*, ACP and OCP). During precipitation, the newly formed crystals grow and form a web of intermingling microneedles or microplatelets of the final products, thus providing mechanical rigidity to the hardened cements. In other words, entanglement of the newly formed crystals is the major reason of setting. For the majority of apatite cements, water is not a reactant in the setting reaction, therefore, the quantity of water actually needed for setting of apatite cements is very small [21,109,111]. However, for brushite cements, water always participates in the chemical transformations because it is necessary for DCPD formation. Due to this reason, brushite cements are always hydraulic, while this term is not usually associated with apatite cements.

Setting of calcium orthophosphate cements is a continuous process that always starts with dissolution of the initial compounds in an aqueous system. This process supplies calcium and orthophosphate ions into the solution, where they interact chemically and precipitate in the form of either the end-products or precursor phases, which causes the cement setting [112,113,114]. This was confirmed by Ishikawa and Asaoka, who showed that when TTCP and DCPA powders were mixed in double-distilled water, both powders were dissolved. The dissolved calcium and orthophosphate ions in the solution were then precipitated in the form of CDHA on the surface of the powders [115]. The precipitate can be either a gel or a conglomerate of crystals. Therefore, the hardening mechanism is either a sol-gel transition of ACP [100] or entanglement of the precipitated crystals of other calcium orthophosphates [25]. For example, for the classical Brown-Chow cement formulation, after the initial setting, petal or needle-like crystals enlarge epitaxially and are responsible for the adherence and interlocking of the crystalline grains, which result in hardening. After ~ 2 hours, the newly formed crystals become rod-like, resulting from higher crystallinity with the observation of more material at the inter-particle spaces. During this period, the cement setting reaction proceeded at a near-constant rate, suggesting that the reaction rate was limited by factors that are unrelated to the amounts of the starting materials and the reaction products present in the system. Such factors could be related to the surface area of DCPA or TTCP or to the diffusion distances over which the calcium and orthophosphate ions migrate in order to form CDHA [116,117,118]. At ~ 24 hours, the crystals are completely formed, being very compacted in some areas of high density and well separated in areas with more porosity [93,98,99].

### 2.3. Chemistry

The chemical reactions that take place during the setting of calcium orthophosphate cements depend on their chemical composition. However, it can be stated that only two major chemical types of setting reactions are possible. The first type occurs according to the classical rules of the acid-base interaction, *i.e.* a relatively acidic calcium orthophosphate reacts with a relatively basic one to produce a relatively neutral compound. The first cement by Brown and Chow is a typical example of this type because TTCP (basic) reacts with DCPA (slightly acidic) in an aqueous suspension to form a precipitated poorly crystalline HA (slightly basic) [14,15]:

2Ca_4_(PO_4_)_2_O + 2CaHPO_4_ → Ca_10_(PO_4_)_6_(OH)_2_ (1)

Earlier, it was believed that DCPA and TTCP reacted upon mixing with water to form the stoichiometric HA [13,14,15,16]. However, further investigations have shown that only the first nuclei consist of a nearly stoichiometric HA, whereas further growth of these nuclei occurs in the form of CDHA [119]. Besides, there is a study demonstrating that the initially formed HA further interacts with remaining DCPD to form CDHA [120].

Formation of HA according to equation (1) releases neither acidic nor basic byproducts. Thus, the liquid phase of the cement remains at a near constant pH of ~ 7.5 for the TTCP + DCPD and ~ 8.0 for the TTCP + DCPA formulations, respectively [116,117,118]. Various deviations from the stoichiometry of chemical equation (1) were studied in details and various apatitic calcium orthophosphates with Ca/P ionic ratio within 1.5 – 1.67 were found as the end-product [121]. The effect of mixing ratio and pH on the reaction between TTCP and DCPA is well described elsewhere [122]. Furthermore, the influence of Ca/P ionic ratio of TTCP on the properties of the TTCP + DCPD cement was studied as well [123].

A blend proposed by Lemaître *et al*. [124,125] is another example of the acid-base interaction where β-TCP (almost neutral) reacts with MCPM (acidic) to form DCPD (slightly acidic):

β-Ca_3_(PO_4_)_2_ + Ca(H_2_PO_4_)_2_·H_2_O + 7H_2_O → 4CaHPO_4_·2H_2_O (2)

In chemical equation (2) MCPM might easily be substituted by orthophosphoric acid [126,127,128,129] or MCPA, while β-TCP might be replaced by either α-TCP [130,131] or CDHA [132,133]. For example:

Ca_9_(HPO_4_)(PO_4_)_5_(OH) + 3H_3_PO_4_ + 17H_2_O → 9CaHPO_4_·2H_2_O (3)

Furthermore, cement formulations based on mixtures of ACP + α-TCP [134], ACP + DCPD [135,136], DCPA + α-TCP [131], OCP + TTCP [137] and partially crystallized calcium orthophosphate + DCPA [138] as the initial reagents, are also available.

The second type of setting reaction might be defined as hydrolysis of a metastable calcium orthophosphate in aqueous media. As the result, both the initial and final compounds have the same Ca/P ionic ratio. Due to the fact, that only one calcium orthophosphate is used; the solid part of such formulations might be called as a single-phase (or single-component) cement powder [139]. Cements made of ACP + an aqueous solution [140,141], α-TCP + an aqueous solution [142,143,144,145,146,147,148], β-TCP + an aqueous solution [146,149], nanocrystalline TTCP + an aqueous solution [150,151] or γ-radiated TTCP + an aqueous solution [152,153,154] are the typical examples; all of them re-crystallize to CDHA upon contact with water:

Ca_x_H_y_(PO_4_)_z_·nH_2_O + H_2_O → Ca_10-*x*_(HPO_4_)*_x_*(PO_4_)_6-*x*_(OH)_2-*x*_ + nH_2_O (4)

3(α- or β-)Ca_3_(PO_4_)_2_ + H_2_O → Ca_9_(HPO_4_)(PO_4_)_5_(OH) (5)

3Ca_4_(PO_4_)_2_O + 3H_2_O → Ca_9_(HPO_4_)(PO_4_)_5_(OH) + 3Ca(OH)_2_ (6)

The experimental details on TTCP hydrolysis under a near-constant composition condition can be found elsewhere [155]. Details on α-TCP hydrolysis are also available. The results indicated that setting of α-TCP was initially controlled by surface dissolution; therefore, it depended on the surface area of the reactants [156,157,158,159]. Hydrolysis of DCPD to CDHA was studied as well [160]. Furthermore, addition of ~ 2 wt. % of a precipitated poorly crystalline HA as a seed to α-TCP powder phase might be useful to accelerate the kinetics of reaction (5) [161].

Further, there is a single-phase cement powder consisting of K- and Na- containing CDHA (with the Ca/P ionic ratio of 1.64 ± 0.02) that sets and hardens after mixing with an aqueous solution of sodium citrate and sodium orthophosphate [17]. After setting, this formulation gives rise to formation of a weak cement (the compressive strength of 15 ± 3 MPa) consisting of the ion-substituted CDHA again (presumably, with a different Ca/P ionic ratio), mimicking the bone mineral. Unfortunately, neither the setting reaction nor the setting mechanism of this cement has been disclosed in literature [17]. What’s more, a self-setting cement might be prepared from the thermal decomposition product of HA [162].

The hydration process of calcium orthophosphate cements is slightly exothermic (which is beneficial for biomedical applications) and takes place in five stages: initiation period, induction period, acceleration period, deceleration period and termination period [163]. For the classical Brown-Chow cement formulation, the activation energy of the hydration reaction is 176 kJ/mol [164]. The rate of heat liberation during the solidification of calcium orthophosphate cements is low. The results of adiabatic experiments showed that the temperature rise arrived at the highest value of 37 °C 3 h later, which would cause no harm to surrounding tissues [163]. The results show that the hardening process of this cement is initially controlled by the dissolution of the reactants in a 4 h period and subsequently by diffusion through the product layer of CDHA around the grains [99]. In general, setting of calcium orthophosphate cements occurs mostly within the initial ~ 6 hours, yielding an ~ 80 % conversion to the final products. The volume of the cements stays almost constant during setting. However, after hardening, calcium orthophosphate cements always form brittle ceramics with the tensile strength of 5 to 20 times lower than the compression strength [165,166]. Since this material is weak under tensile forces, these cements can only be used either in combination with metal implants or in non-load bearing (*e.g.*, craniofacial) applications [111,167,168,169]. This is confirmed by the mechanical characterization of a bone defect model filled with ceramic cements [170].

To conclude this part, one must stress, that chemical equations (1) – (6) of the cement setting are valid for the *in vitro* conditions only. There are evidences that samples of calcium orthophosphate cement retrieved 12 h after hardening *in vivo* already contained carbonateapatite, even though the initial cement mixture did not contain carbonate as one of the solid components [171]. The mass fraction of carbonate in the 12 h samples was about 1 %. The results suggest that under the *in vivo* conditions, carbonate is readily available and this allows formation of carbonateapatite in favor of carbonate-free CDHA [171].

### 2.4. Market

**Figure 1:**
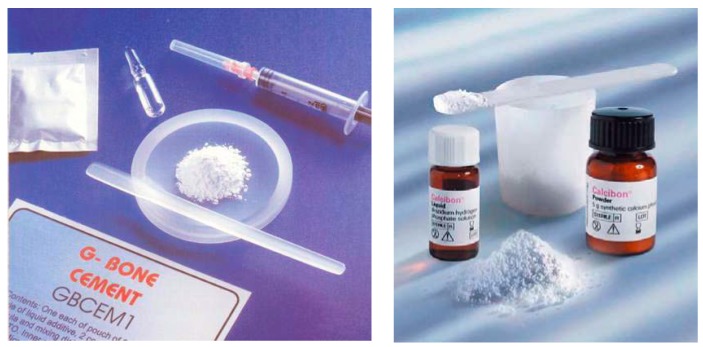
A presentation of two randomly chosen commercial calcium orthophosphate cements.

The United States Food and Drug Administration (FDA) has approved several cement formulations for clinical use [21,172]. Some examples are given in Table 2. The same formulations have also received a Conformite Europene (CE) mark for certain maxillofacial indications and for use as a bone-void filler in the specific non-load-bearing orthopedic indications [111]. The major properties of these formulations are available in literature [21]. The list of other commercially available injectable bone cements with their chemical composition (when obtainable) might be found elsewhere [30,118,173,174], while the various types of bone cements and fillers are listed in another review [168]. Besides, many more cement formulations are in experimental stages. A general appearance of two randomly chosen commercial calcium orthophosphate cements is shown in Figure 1.

**Table 2:** Some calcium orthophosphate cement formulations having the 510(k) clearance from the FDA [20,111,172]. The technical data on these cements might be found in literature [21].

Product^*^	Manufacturer	Applications^*^
BoneSource^TM**^	Striker Howmedica Osteonics (Rutherford, NJ)	Craniofacial
α-Bone Substitute Material (α-BSM^®^)^***^	Etex Corporation (Cambridge, MA)	Filling of bone defects and voids, dental, craniofacial
Skeletal Repair Systems (SRS^®^)	Norian Corporation (Cupertino, CA)	Skeletal distal radius fractures, craniofacial

^*^ In Europe, other applications may apply, and the materials may be sold under a different commercial name. ^**^ BoneSource^TM^ is the original formulation of calcium orthophosphate cement developed by Brown and Chow. ^***^ In Europe, it is distributed by Biomet Merck (Zwijndrecht, The Netherlands) as Biobon^®^ [111], while in North America it is marketed by Walter Lorenz Surgical (Jacksonville, FL) as Embarc^®^ [21].

## 3. Two Major Types of Calcium Orthophosphate Cements

### 3.1. Apatite Cements

Typically, apatite cement formulations have a precipitated poorly crystalline HA and/or CDHA as the end-product of the setting reaction [see chemical equations (1), (4) – (6)], although traces of the unreacted starting materials can be present [93]. Due to the initial presence of carbonates, apatite cements such as Norian SRS^®^ and Biocement D^®^ form a non-stoichiometric carbonatapatite or dahllite [Ca_8.8_(HPO_4_)_0.7_(PO_4_)_4.5_(CO_3_)_0.7_(OH)_1.3_] as the end-product [45,175]. As both CDHA and carbonateapatite are formed in an aqueous environment and have a low crystallinity, they appear to be similar to biological apatite of bones and teeth. These properties are believed to be responsible for the excellent *in vivo* resorption characteristics. Conventional apatite cements contain TCP and/or TTCP phases in their powder components [30], while a single component cement powder consisting of K- and Na- containing CDHA is also available [17]. The reactivity of TCP-based apatite cements varies as a function of TCP crystal phase, crystallinity and particle size [176,177]. Generally, a higher reactivity is observed with a thermodynamically less stable phase (from β-TCP to α-TCP and ACP) and with a smaller particle size [146]. Nominally, it might be stated that formation of apatites through a cementation reaction is a sort of a biomimetic process because it occurs in physiological environment and at body temperature [33]; however, both the crystallization kinetics and a driving force are very far away from the biomimeticity. A unique feature of the hardened apatite cements is that the force linking the newly formed crystals (of both CDHA and carbonatapatite) is weak; therefore, the crystals can be easily detached from the cement bulk, especially after dissolution has partly occurred. When this happens, osteoclasts and other cells can easily ingest the apatite crystals [178].

Immediately after implantation, any cement becomes exposed to blood and other tissue fluids that delay the setting time. Intrinsic setting time for apatite cements has been extensively studied and it appeared to be rather long. For example, for the original formulation by Brown and Chow it ranged from 15 to 22 min [14,15]. This may result in procedural complications. To remedy this, the amount of liquid might be reduced to a possible minimum. Therefore, all apatite cements are viscous and easily moldable pastes but tend to be difficult to inject. Besides playing with the P/L ratio, the setting time can also be reduced by using additives to the liquid phase (which in the Brown-Chow formulation is distilled water [14,15]). The list of additives includes phosphoric acid, MCPM and other soluble orthophosphates. These additives promote dissolution of the solids by lowering the solution pH. In such cases, a setting time in the range of 10 – 15 minutes can be obtained [140,142,143,144,145,146,147,148,179]. The influence of soluble orthophosphates (*e.g.,* Na_2_HPO_4_ or NaH_2_PO_4_) on the setting time of apatite cements is explained by the fact that dissolution of DCPA and formation of CDHA during setting occur in a linear fashion, thus avoiding early formation of CDHA. This is important because too early formation of CDHA might engulf un-reacted DCPA, which slows down DCPA dissolution and thus the setting kinetics becomes slower, while the presence of sodium orthophosphates prevents DCPA particles from being isolated [180]. Particle size [161,181,182], temperature of the liquid phase and initial presence of HA as a seed in the solid phase are other factors that influence the setting time [14,15,33,176,177]; however, *in vitro* studies demonstrated that these parameters did not affect significantly [93]. On the other hand, a reduction in particle size was found to result in a significant decrease in both initial and final setting times [161,181,182], an acceleration of the hardening rate [161] and hydration kinetics of the hardening cement [182]. Besides, the crystallite sizes of the final product can be strongly reduced by increasing the specific surface of the starting powder, which allows developing calcium orthophosphate cements with tailored structures at the micro and nanoscale levels [161]. Unfortunately, an unclear correlation was found between the particle dimensions of the initial calcium orthophosphates and mechanical properties of the hardened cements: namely, a significant increase in compressive strength and storage modulus was reported for some formulations [181,182] but a minor effect on compressive strength was discovered for other ones [161]. This inconsistence is not surprising because the manufacturing method used to produce test samples varies from one author to the other. Therefore, the only remaining fact is that calcium orthophosphate cements are brittle and hence worthless for load-bearing applications [167,168].

Setting process of the most types of apatite cements occurs according to just one chemical reaction (see chemical equations (1), (4) – (6)) and at near the physiological pH. The latter may additionally contribute to the high biocompatibility observed for these materials [116,117,118]. For the classical formulation by Brown and Chow, the transmission electron microscopy results suggested the process for early-stage apatite formation as follows: when TTCP and DCPA powders were mixed in an orthophosphate-containing solution, TTCP powder was quickly dissolved due to its higher solubility in acidic media. Then the dissolved ions of calcium and orthophosphate, along with ions already existing in the solution, were precipitated predominantly onto the surface of DCPA particles. Few apatite crystals were observed on the surface of TTCP powder. At a later stage of the reaction, an extensive growth of apatite crystals or whiskers effectively linked DCPA particles together and bridged the larger TTCP particles causing the cement setting [183].

However, Norian SRS^®^ and Cementek^®^ were found to set according to two chemical reactions: precipitation of DCPD, followed by precipitation of either CDHA or carbonatapatite:

α-Ca_3_(PO_4_)_2_ + Ca(H_2_PO_4_)_2_·H_2_O + 7H_2_O → 4CaHPO_4_·2H_2_O (7)

5.2CaHPO_4_·2H_2_O + 3.6CaCO_3_ → Ca_8.8_(HPO_4_)_0.7_(PO_4_)_4.5_(CO_3_)_0.7_(OH)_1.3_ + 2.9CO_2_ + 12H_2_O (8)

The initial chemical reaction (7) was very fast and provoked DCPD formation and setting of the cement pastes within seconds. The second step was slower: DCPD reacted completely within several hours with remaining α-Ca_3_(PO_4_)_2_ and CaCO_3_ forming carbonatapatite according to equation (8). The latter step caused the cement hardening. A similar two-step hardening mechanism was established for a cement consisting of MCPM and CaO: in the first step, during the mixing time, MCPM reacted with CaO immediately to give DCPD, which, in the second step, reacted more slowly with the remaining CaO to give CDHA [50].

The aforementioned setting mechanism of an apatite cement was investigated in details for a three component mixture of TTCP, β-TCP and MCPM dry powders in convenient proportions and with the overall atomic Ca/P ratio equal to 1.67. Two liquid phases in a raw were used to damp the cement powder, initially it was water + ethanol (ethanol was added to slow down the hardening) and afterwards orthophosphoric acid and sodium glycerophosphate were added to water to prepare a reactive liquid [109]. At the very beginning, DCPD was found to form according to two chemical reactions:

Ca(H_2_PO_4_)_2_·H_2_O + β-Ca_3_(PO_4_)_2_ + 7H_2_O → 4CaHPO_4_·2H_2_O (9)

Ca_4_(PO_4_)_2_O + 2H_3_PO_4_ + 7H_2_O → 4CaHPO_4_·2H_2_O (10)

The formation reactions of DCPD were fast and corresponded to the setting stage. Afterwards, TTCP reacted with the previously formed DCPD and with β-TCP to give CDHA according to the reactions:

2Ca_4_(PO_4_)_2_O + 2CaHPO_4_·2H_2_O → Ca_10-*x*_(HPO_4_)*_x_*(PO_4_)_6-*x*_(OH)_2-*x*_ + *x*Ca(OH)_2_ + (4 – *x*)H_2_O (11)

2Ca_4_(PO_4_)_2_O + 4β-Ca_3_(PO_4_)_2_ + (2+2*x*)H_2_O → 2Ca_10-*x*_(HPO_4_)*_x_*(PO_4_)_6-*x*_(OH)_2-*x*_ + 2*x*Ca(OH)_2_ (12)

The formation reactions of the CDHA phase were quite slow and corresponded to the hardening stage. Although OCP was not detected in that study, its formation as an intermediate phase was postulated for this cement [109]. A similar suggestion on the intermediate formation of OCP was made for the setting mechanism of Brown-Chow classical cement formulation [88,93]; however, a reliable evidence for its presence is still lacking [143,184]. In both cases, OCP was suggested to appear as an intermediate because it was a faster forming phase than CDHA. This hypothesis is based upon the classical studies performed by Prof. W. E. Brown *et al*. about the precursor phase formation during chemical crystallization of apatites in aqueous solutions [185,186,187].

Solubility of the hardened apatite cements in aqueous solutions is expected to be rather similar to that of bone mineral. This means that they are relatively insoluble at neutral pH and increasingly soluble as pH drops down; this is an important characteristic of normal bone mineral that facilitates controlled dissolution by osteoclasts [175].

To conclude this part, one should mention, that in 2000 the US bone substitute market for Norian SRS^®^ accounted for ~ 15 % of the total sales, followed by BoneSource^TM^ at ~ 13 %, and α-BSM^®^ at ~ 8.5 % [111].

### 3.2. Brushite Cements

As indicated by its name, DCPD is the major end-product of the setting reaction of brushite cements (chemical equations (2) and (3)). Mirtchi and Lemaître [124] and independently Bajpai *et al*. [125] introduced this type of the cements in 1987. To date several formulations have been already proposed, *e.g.,* β-TCP + MCPM [124,126], β-TCP + H_3_PO_4_ [125,127,128] and TTCP + MCPM + CaO [188]. All brushite cements are set by the acid-base interaction only. As DCPD can only precipitate at the solution pH < 6, the paste of brushite cement is acidic during setting [127,189]. For example, during setting of a β-TCP + MCPM cement, the cement pH varies from very acidic pH values of ~ 2.5, to almost neutral pH values of ~ 6.0 [127]. Replacing MCPM by orthophosphoric acid renders the cement paste very acidic for the initial ~ 30 s but then the pH profile follows that obtained with MCPM. It is important to notice, that β-TCP + H_3_PO_4_ formulations have several advantages over β-TCP + MCPM formulations, namely: (i) easier and faster preparation, (ii) a better control of the chemical composition and reactivity, (iii) improved physico-chemical properties, such as longer setting times and larger tensile strengths due to a higher homogeneity. However, the use of orthophosphoric acid might impair the biocompatibility of the cement formulation, due to low pH values during setting [127]. If a cement paste contains an excess of a basic phase, the equilibrium pH will be given by the intersection of the solubility isotherms of the basic phase with that of DCPD. For example, the equilibrium pH values of β-TCP + MCPM, HA + MCPM and TTCP + MCPM mixtures are 5.9, 4.2 and 7.6, respectively [167,168].

As the solubility of calcium orthophosphates decreases with increasing of their basicity (Table 1), the setting time of brushite cements much depends on the solubility of a basic phase: the higher its solubility, the faster the setting time. Therefore, the setting time of the cements made of MCPM + a basic calcium orthophosphate increases in the order: HA > β-TCP > α-TCP [167,168]. For example, HA + MCPM mixtures have a setting time of several minutes, β-TCP + MCPM mixtures – of 30 to 60 seconds and α-TCP + MCPM mixtures – of a few seconds [124,125]. Despite this initial high reactivity, the hardening reaction of brushite cements typically lasts one day until completion [176,177]. Additives that inhibit the crystal growth of DCPD have successfully been used to increase the setting time of β-TCP + MCPM mixtures [190]. In contrast to apatite cements, brushite cements can be initially liquid and still set within a short period of time [167,168].

Brushite is remarkably biocompatible and bioresorbable [189]. Due to both a better solubility of DCPD if compared to that of CDHA (Table 1) and metastability of DCPD under physiological conditions [191], brushite cements degrade faster than apatite ones [192,193,194]. They are quickly resorbed *in vivo* and suffered from a rapid decrease in strength (although the overall mechanical properties of the healing bone increase as bone ingrowth occurs [31]). Short setting times, low mechanical strength and limited injectability seem to prevent brushite cements from a broader clinical application. However, the major reason why brushite cements are not more widespread is probably not related to the mechanical issues but just to a later arrival on the market. Use of sodium citrate or citric acid as setting retardants is an option to get more workable and less viscous pastes of brushite cements [22,195,196,197,198]. Similar effect might be achieved by addition of chondroitin 4-sulfate [199] and glycolic acid [200]. For the cement formulations with orthophosphoric acid as the initial reactant (see chemical equation (3)), acid deficient formulations were also found to improve the workability. In this case, the setting reaction might be described by the following chemical equation [198]:

3.7β-Ca_3_(PO_4_)_2_ + H_3_PO_4_ + 27.8H_2_O → 3CaHPO_4_·2H_2_O + 2.7β-Ca_3_(PO_4_)_2_ + 21H_2_O (13)

Although, several studies revealed that too much of DCPD in a given volume was not detrimental to the biological properties of brushite cements [31,175,188], occasionally, when large quantities of brushite cements were used, a certain degree of tissue inflammation during the first weeks of *in vivo* implantation were reported [194,198,201]. Further investigations indicated that the inflammatory could be due to a partial transformation of DCPD into CDHA with release of orthophosphoric acid [202]:

(10 – *x*)CaHPO_4_·2H_2_O → Ca_10-*x*_(HPO_4_)*_x_*(PO_4_)_6-*x*_(OH)_2-*x*_ + (4 – *x*)H_3_PO_4_ + (18 – *x*)H_2_O (14)

Transformation of DCPD into CDHA occurs via two successive processes: dissolution and precipitation [203] and can be retarded by adding magnesium ions to the cement paste, thus reducing the possibility of inflammation [167,168]. The aforementioned case of acid deficient formulations of brushite cements (chemical equation (13)) is an alternative, because it reduces the amount of unreacted acid in the cement [198] with an option to consume liberating in chemical equation (14) orthophosphoric acid by the excess of β-TCP. Implantation of previously set brushite cement might be the third option, because a solid material was found to be better tolerated than paste implants. Besides, more bone was formed at the solid implant contact and the solid material degraded not so rapidly [204]. For brushite cements, a linear degradation rate of 0.25 mm/week was reported [205]. This rapid degradation rate might lead to formation of an immature bone. Adding β-TCP granules to the cement paste could solve this problem because β-TCP granules might act as bone anchors and encourage formation of a mature bone [205,206].

## 4. Various Properties of Calcium Orthophosphate Cements

### 4.1. Setting and Hardening

Generally, calcium orthophosphate cements must set slowly enough to provide sufficient time to a surgeon to perform implantation but fast enough to prevent delaying the operation. Ideally, good mechanical properties should be reached within minutes after initial setting. Two main experimental approaches are used to study the cement setting process: a batch approach and a continuous approach. In the batch approach, the setting reaction is stopped at various times and the resulting samples are analyzed to determine *e.g.,* the composition and compressive strength of the samples [176,177]. There are currently two standardized methods to apply this approach, namely, the Gillmore needles method (ASTM C266-89) [207] and the Vicat needle method (ASTM C191-92) [208]. The idea of both methods is to examine visually the surface of cement samples to decide whether the cement has already set, *i.e*. if no mark can be seen on the surface after indentation. Besides, the setting process might be monitored in real time by non-destructive methods (the continuous approach), *e.g.*, pulse-echo ultrasound technique [209,210], isothermal differential scanning calorimetry [145,146,211,212,213,214,215,216] and alternating current (AC) impedance spectroscopy [217]. For example, recent calorimetry measurements suggested that in equation (2) the endothermic MCPM dissolution and the highly exothermic β-TCP dissolution occurred simultaneously, followed by the exothermic crystallization of DCPD [215]. Moreover, acid-base reactions (1) – (3) can be and have been analyzed by measuring the pH evolution of a diluted cement paste [176]. Finally yet importantly, methods of Fourier-transform infrared spectroscopy [216,218], X-ray diffraction [43,130,219] and energy dispersive X-ray diffraction [220] might be applied as well. The latter techniques proved to be powerful, even though they have limitations such as the time required for each measurement (the 250 s required for an X-ray diffraction scan is a problem for fast setting reactions); besides the analysis is located at the surface of the sample where evaporation and thermal effects can modify the reaction rate of the surface compared to that of the bulk. Furthermore, the continuous approach is an indirect one, which markedly complicates an interpretation of the collected data, particularly in complex cement formulations [176].

**Figure 2:**
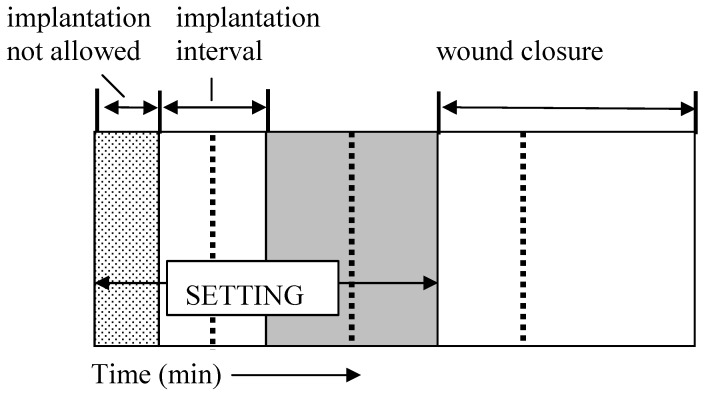
A diagram of the setting parameters relevant for a calcium orthophosphate cement: *CT* – cohesion time; *I* – initial setting time; *F* – final setting time. Adapted from Ref. [25] with permission.

A way to assess the rate of a cement hardening is to measure its setting time, which means the time required to reach a certain compressive strength, generally close to 1 MPa. The most straightforward approach is to prepare cement samples with a well-controlled geometry (*e.g.,* cylinders), incubating these samples for various times in the right environment (temperature, humidity) and assessing the composition and mechanical properties of the samples as a function of time [176]. One should stress, that setting time for calcium orthophosphate cements often corresponds to an earlier stage in the overall setting reaction, typically 5 – 15 % of the overall reaction, while the end of the cement setting is typically reached after several days [93,143]. Gillmore needles have been used with success to measure the initial (*I*) and final (*F*) setting times of calcium orthophosphate cements [86]. A light and thick needle is used to measure the initial setting time *I*, while a heavy and thin needle for the final setting time *F* [108]. The clinical meaning is that the cement paste should be implanted before time *I* and that the wound can be closed after time *F* (Figure 2).

The cement should not be deformed between times *I* and *F* because in that stage of the setting process any deformation could induce cracks [25]. The following handling requirements (in minutes) have been formulated for calcium orthophosphate cements, as a result [108,221]:

3 ≤ *I* < 8

*I* – *CT* ≥ 1

*F* ≤ 15

These parameters are represented schematically in Figure 2. The second requirement means, that the cohesion time (*CT*) must be at least 1 min before *I*, so that a clinician has at least 1 min to apply and to mold the material. *CT* is the time from which a cement no longer disintegrates when immersed in Ringer’s solution [108]. As the mixing in a mortar is about 1 min, the shortest *CT* that can be allowed is about 2 min, so that a clinician has at least 1 min to collect the paste from the mortar and put it on the pallet knife or in the syringe with which it is to be transferred to the wound after *CT* and before *I* [108]. For dental applications, time *I* must be close to 3 min, whereas for orthopedic applications it must be close to 8 min. However, in no case it will be tolerable for the clinicians if time *F* becomes greater than 15 min [25,108].

### 4.2. Handling

In the clinical situation, calcium orthophosphate cements can be either applied by the fingertips of a surgeon or injected from a syringe to the defect area of a bone. The first type of clinical application requires formulation of a high-viscosity cement paste, which can be applied manually as dough, while the second type of clinical application requires formulation of a low-viscosity cement paste, which can be applied by injection from a syringe [108]. Currently, injection appears to be the preferred method between these two major options. Thus, a trade-off must be found between a high viscosity leading to too high injection forces and a low viscosity increasing the risk of cement extravasations. Viscosity values in the range of 100 – 2,000 Pa·s are generally considered to be adequate [222].

In any case, before using a surgeon needs to have a cement powder and a liquid be mixed properly and thoroughly (to avoid the powder/liquid encapsulation) within the prescribed time and be performed in a sterile environment. Therefore, a mixing procedure is very important because prior to be injected, a cement paste must be transferred from a mixing chamber into a syringe. Ideally, this should be done without trapping of air bubbles by the cement paste [223]. Earlier, most calcium orthophosphate cements were manually mixed with aqueous solutions using a mortar and either a pestle or a spatula. At the time, some concerns were raised about an insufficient and inhomogeneous mixing thus compromising the implant strength, as well as on inconsistencies between operators causing unpredictable variations in graft performance [224]. Mechanical mixing (*e.g.*, by either an electrically powered mixing machine of Norian SRS/CRS^®^ or Mini-malax^®^ mixing system for Cementek^®^ cement, produced by Teknimed S.A.) is the modern approach. It allows mixing the cement paste within 60 – 80 s and enables a rapid and reliable filling of the application syringe [30]. Besides, a cement powder and an aqueous solution might be placed into a syringe and mixed inside a shaker to produce a consistent cement paste of the desired viscosity [223]. The mechanical mixing was found to decrease both the mean viscosity of the curing cement paste and variability in the viscosity at a given time [225]. However, it did not improve the mechanical strength of the cement [167,168].

Of the cements listed in Table 2 Norian SRS^®^ is sold as a reactant pack containing two components: a mixture of dry powders (MCPM + α-TCP + CaCO_3_) and a liquid (aqueous solution of Na_2_HPO_4_). The components are mixed in the operating room. The paste that is formed is malleable and injectable for ~ 5 minutes; it hardens within ~ 10 minutes after injection [21,175]. However, data are available that out of 4.5 mL Norian SRS^®^ cement paste only ~ 3 mL is injectable, whereas up to 1.5 mL of the cement might remain uninjectable from the syringe [25]. This phenomenon is prescribed to the cement rheology and its interaction with the hydraulic forces of the syringe. α-Bone Substitute Material (α-BSM^®^) is also a two-component system; it is prepared from a mixture of ACP and DCPD powders and a saline solution [140]. Biopex^®^ consists of four different calcium orthophosphates: 75 wt. % α-TCP, 18 wt. % TTCP, 5 wt. % DCPD and 2 wt. % HA. The aqueous solution contains 12 wt. % sodium succinate and 5 wt. % sodium chondroitin sulfate [226]. Effects of liquid phase on the basic properties of Biopex^®^ were investigated. When mixed with neutral sodium hydrogen orthophosphate or succinic acid disodium salt solution, the initial setting times of the cement were 19.4 ± 0.55 and 11.8 ± 0.45 minutes respectively. These setting times were much shorter than that of distilled water, 88.4 ± 0.55 minutes [227]. Biopex^®^ is mixed with a spatula inside a syringe that can be opened from the front. After mixing, the front part is closed, a needle is inserted into this front part and the cement paste can be manually injected [167,168].

### 4.3. Physical and Rheological Properties

Some systematic studies on the influence of composition and concentration of the liquids used in preparing of calcium orthophosphate cements were performed as well [22,195]. Unfortunately, the results appeared to be rather unclear. For example, for several cements, mixing with sodium citrate or citric acid resulted in some effects on the initial setting time [22,196], while for other cements the effect was insignificant [195]. Concentration increasing of sodium citrate solution resulted in initial setting time increasing [22,195], although the injectability variations of the cement pastes were inconsistent [22,196].

**Figure 3:**
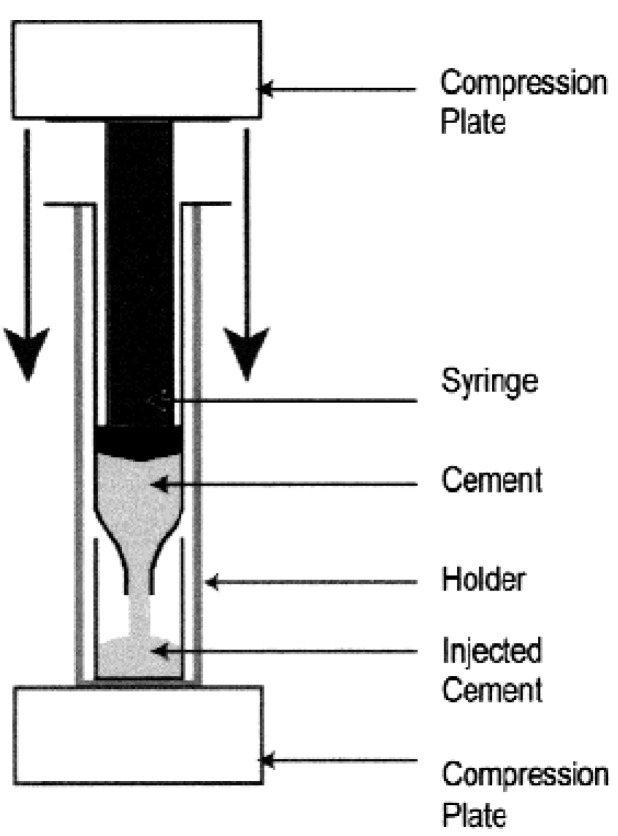
A schematic representation of the experimental setup used to quantify the injectability of the calcium orthophosphate cements. Reprinted from Ref. [234] with permission.

Good injectability, adequate viscosity and satisfactory cohesion are required for the successful biomedical applications of calcium orthophosphate cements [228,229]. Injectability is the ability to be extruded through a small hole of a long needle (*e.g.*, 2 mm diameter and 10 cm length) [230,231] (other needles are also applied [232,233]); and for certain applications, injectability is even a prerequisite. It is measured by the weight percentage of the cement paste that could be injected without demixing from a standard syringe by either a hand or a force of 100 N maximum (Figure 3).

Usually, injectability of a cement paste varies inversely with its viscosity, the P/L ratio, as well as the time after starting the mixing of liquid and powder [48,231,234]. When put under pressure, some calcium orthophosphate cements show demixing into a thin paste, which is extruded, and a thick mass, which remains inside the syringe (see the aforementioned example for Norian SRS^®^ [25]). The phenomenon, in which the pressure applied to the cement paste provokes a phase separation after a certain injection time, is called filter pressing: the liquid comes out without the particles [230]. Possible mechanisms underlying the limited injectability of hydraulic calcium orthophosphate cements have been discussed in literature [233]. In the case of demixing, the exact composition of the extruded part of the paste becomes unknown. Moreover, due to a deviation from the initial P/L ratio, it becomes unclear whether the setting behavior and the mechanical and histological properties of the extruded part are still clinically acceptable. Therefore, a good cohesion of the paste is necessary in order to avoid these problems [235].

An appropriate cohesion was achieved when no disintegration of the cement paste was observed in the fluid [108,235]. This can be accomplished by keeping a high viscosity for the cement paste [21] or using cohesion promoters (*e.g.*, 1 % aqueous solution of sodium alginate [147,236,237] and other chemicals [147,238]). Some calcium orthophosphate cements fulfill both criteria, *e.g.,* Norian SRS^®^, but others fulfill only one or even none of these requirements. For example, BoneSource^TM^ [90] and Cementek^®^ are not injectable and blood must be kept away from the implanting site until setting [167,168]. A poor cohesion has been associated to a poor biocompatibility and might lead to inflammatory reactions [239]. Further details on the cohesion properties of various calcium orthophosphate pastes are available in literature [235].

Several ways can be adopted to improve both the *in vitro* properties and behavior of calcium orthophosphate cements. The first approach consists of injectability improvement. There are two options for this. Firstly, the injection device can be modified. For example, shorter cannulas with a larger diameter, as well as smaller injection rates favor a good injectability. The last option is not so straightforward: for example, Habib *et al*. have shown that large injection rates are not detrimental to injectability because of the shear-thinning behavior of many calcium orthophosphate cements [233]. Secondly, the cement composition can also be adapted. Namely, a decrease of the particle size, the P/L ratio and the plastic limit were found to contribute to a better injectability [230,234]. For example, injectability was found to be unaffected by P/L ratio within the range of 3.85 – 4.50 g/mL but drops by nearly 100 % between P/L ratio of 4.50 and 5.00 g/mL [22]. However, a decrease in P/L ratio leads to a decrease in the mechanical properties of the cements and the cohesion might be destroyed. Furthermore, both the initial and final setting times decreased markedly with the P/L ratio increasing [195,240]. Therefore, variations in the P/L ratio appear to be valid to a certain extent only. That is why the manufacturer of Biopex^®^ suggests using a P/L ratio of 2.8 or 3.3 g/mL.

Decreasing the particle size of calcium orthophosphate crystals is the second approach for the injectability improvement. For example, α-BSM^®^ is well injectable because it consists of small particles. Even though small particles require a larger amount of mixing liquid to obtain a paste, injectability and cohesion of the cements are generally very good [167,168]. An indirect approach is to add calcium orthophosphate particles those act as spacers between other particles. For example, DCPA is added to the formulation of Biocement D^®^ to improve the injectability of the paste [167,168]. Similarly, there is an apatite cement containing spherical particles of TTCP to improve injectability [241].

### 4.4. Using Additives

Using various additives is the second way to improve the physical properties of calcium orthophosphate cements [242]. For example, water demand of calcium orthophosphate cements can be reduced by ionically modifying the liquid component, *e.g.,* by adding nontoxic sodium salts of α-hydroxy di- and tri- acids [243,244]. A list of additives, that have been already studied, includes fluidificants, air-entraining agents, porogens, workability-improvement agents, setting time controllers and reinforcing additives [135,173,245]. Besides, radiopacifiers might be used [246]. The main role of a fluidificant is to reduce a mixing time of the cement. Citric acid is an example of such a reagent; it retards the dissolution-precipitation reactions in the cement, decreases the compressive strength during initial setting, but increases its strength in the final stages of the cement hardening [196]. Furthermore, data are available indicating that citric acid decreases the setting time and improves the mechanical properties of the hardened cements [247]. Air-entraining agents (*e.g.,* surfactants) are commonly used to induce macroporosity inside calcium orthophosphate cements without affecting their normal setting. For example, crystals of mannitol, CH_2_OH(CHOH)_4_CH_2_OH, were tested as an air-entraining agent; however, both loss of workability during the cement mixing and severe depreciation of mechanical properties were discovered simultaneously [248]. Various porogenic agents (*e.g.*, oxygen peroxide [249] in the liquid phase and/or iced [250], sucrose granules, NaHCO_3_ and Na_2_HPO_4_ crystals of 125-250 µm in size [251], poly(DL-lactic-*co*-glycolic acid) microparticles with the average size of 66 ± 25 µm [252,253,254,255,256,257], calcium sulfate [40], NaCl crystals varying in size from 420 μm to 1 mm [258,259], gelatin microspheres [260,261], cetyltrimethyl ammonium bromide [262], polytrimethylene carbonate [263], some immiscible liquids) have been also tested to create porosity. These additives could be applied on pre-set cements only. After cement hardening, dissolution of the aforementioned soluble porogens in either water or body fluids produces macropores with the dimensions and shapes of the dissolved crystals. Another method consisting in adding solid NaHCO_3_ to the starting cement powder and using two different liquids: first, a basic liquid to form the paste and later an acid liquid to obtain CO_2_ bubbles to create the porosity is also available [264]. Besides, pore forming CO_2_ bubbles appear at hardening of an apatite cement, consisting of an acidic calcium orthophosphate and either CaCO_3_ [29,45,46,47] or NaHCO_3_ [265,266,267]. Furthermore, addition of an effervescent porogen formulation comprised from NaHCO_3_ (54.52 %) and citric acid monohydrate (45.48 %) has been suggested [268]. Adding of surfactants to calcium orthophosphate cements was found to have two different functions: they might act as both air-entraining agents by lowering the surface tension [269] and as interaction modifiers by shifting the isoelectric point [270].

The major examples of workability-improvement agents, which are added to the cement powders, include water-soluble polymers. Specifically, polysaccharides [84,95,271,272,273,274], gelatin [240,275,276,277,278,279,280,281] and polyacrylic acid [282,283,284] are of interest due to their biocompatibility and good rheological properties. Only small amounts (a few weight %) are needed to dramatically increase the viscosity of the resulting cement pastes. Besides, the cement paste becomes more cohesive and highly resistant to washout immediately after mixing. For example, a 5 wt. % sodium chondroitin sulfate solution is used as mixing liquid in Biopex^®^ [167,168]. In the case of gelatin, more than a 50 % improvement of the compressive strength was detected [277]. The gelatin-cement after setting was found to exhibit reduced crystallinity, much smaller CDHA crystals and a more compact microstructure; all these phenomena might be accounted for the improved mechanical properties [278]. The presence of gelatin improved mechanical properties of the cements; in particular, calcium orthophosphate cements containing 2 wt. % gelatin were found to harden in an acceptable time and were recommended for clinical applications [281]. The use of gelling agents widened a possible application of calcium orthophosphate cements because these cements can be used even when complete homeostasis is difficult. In some cases addition of a gelling agent might cause an increase in hardening time but this was remedied by the use of a sodium orthophosphate solution as the cement liquid [117,118]. Most polysaccharide solutions are thixotropic, *i.e.*, the viscosity of the solution decreases as the shear rate increases. Certain polysaccharides, such as sodium alginate, pectize in contact with calcium ions. This property can be used to make putty-like cement pastes [21]. However, only a few polysaccharides are accepted for parenteral use [167,168].

Of the two families of calcium orthophosphate cements, the brushite cements react generally much faster than apatite ones. As a result, to satisfy the clinical requirements (Figure 2), the setting time of brushite cements has to be prolonged, whereas that of apatite cements has to be shortened [167,168]. In general, setting reactions of any calcium orthophosphate cements consist of three successive stages: (1) dissolution of reactants to saturate the mixing liquid in calcium and orthophosphate ions; (2) nucleation of crystals; (3) growth of crystals. Therefore, experimental approaches to modify the setting reaction of calcium orthophosphate cements are to be targeted to these three stages. The available approaches have been summarized in Table 3 [176].

**Table 3:** List of strategies and approaches to modify reactivity of calcium orthophosphate cements [176].

Strategy	Approach	Sub-approaches
1. Dissolution rate	1.1. Change contact area between reagent and mixing liquid	1.1.1. Change milling duration
		1.1.2. Use nano- or micro-sized powders
	1.2. Change solubility in the mixing liquid	1.2.1. Use more/less soluble phase
		1.2.2. Change of reaction pH
	1.3. Change saturation of the mixing liquid	
	1.4. Use dissolution inhibitors in the mixing liquid	
	1.5. Modify reagent surface	1.5.1. Chemical change (pre-reaction)
		1.5.2. Physical change (dissolution pits)
2. Nucleation rate	2.1. Use crystallization nuclei	
	2.2. Change the saturation of the reaction product in the mixing liquid	2.2.1. Change of saturation
		2.2.2. Change of end-product solubility
	2.3. Use nucleation inhibitors	
3. Growth rate	3.1. Change the saturation of the reaction product in the mixing liquid	3.1.1. Change of saturation
		3.1.2. Change of end-product solubility
	3.2. Use crystal growth inhibitors	

Furthermore, seven strategies have been described to control the setting time of calcium orthophosphate cements [177]. They are: (i) mean particle size decreasing of the initial powders; (ii) the P/L ratio increasing; (iii) pH drop of the mixing liquid to increase calcium orthophosphate solubility and hence accelerate the chemical transformations; (iv) a nucleating phase addition, such as a nanosized HA powder; (v) adding orthophosphate and/or calcium ions into the mixing liquid to accelerate the setting reaction according to the common-ion effect; (vi) solubility reducing of the reaction end-product, for example, by adding fluoride ions into the mixing liquid; (vii) solubility increasing of the starting material by amorphization, *e.g.,* by prolonged milling. For further details on these strategies and approaches, as well as for application examples, the interested readers are referred to the original papers [176,177].

Various setting time controllers (accelerators and retardants) are used to influence the setting time. They include sodium hydrogen pyrophosphate (Na_2_H_2_P_2_O_7_) and magnesium sulfate (according to another study, ions of citrate, sulfate and pyrophosphate are necessary [190]), which are added in amounts < 1 wt. % [285]). Application of biocompatible α-hydroxylated organic acids (glycolic, lactic, malic, tartaric and citric acids) and their calcium and sodium salts for the modification of both rheological and setting properties of calcium orthophosphate cements is well described elsewhere [286,287]. Besides, aqueous solutions of sodium orthophosphates are also known as setting time accelerators [84,180,256,288,289,290,291]. An extensive list of the compounds, which might be suitable as accelerators, retarders, additives or reactants in calcium orthophosphate cement formulations, may be found in the literature [86]. The subject of the reinforcing additives is discussed in details below in “Reinforced calcium orthophosphate cement composites and concretes” section.

The factors that significantly influenced the storage stability (shelf life) of initial dry powders of calcium orthophosphate cements, were found to be temperature, humidity and the mixing regime of the powders. Various storage conditions appeared to be effective in prolonging the stability of dry brushite cements; in the order of effectiveness, they were ranked as: adding solid citric acid retardant > dry argon atmosphere ≈ gentle mixing (minimal mechanical energy input) >> low temperature [289]. A detailed description of the sterilization techniques for calcium orthophosphate cements can be found elsewhere [292].

## 5. Bioresorption and Replacement of the Cements by Bones

Due to the excellent bioresorbability of DCPD and CDHA, a newly forming woven bone might substitute the hardened calcium orthophosphate cements. For example, implants made of BoneSource^TM^ were partly resorbed and replaced by natural bone, depending upon the size of the cranial defect [90]. α-BSM^®^ was evaluated in a canine femoral slot model. New bone was found to form in three weeks via an osteoconductive pathway. After four weeks, only 1.7 % of the implanted material was observed. The hybrid bone possessed the strength of normal, unoperated bone after 12 weeks. In 26 weeks, the boundary between old and new bone was virtually indistinguishable, with only 0.36 % of the implant recognizable [140]. Norian SRS^®^ was evaluated in canine tibial and femoral metaphyseal defects. The cement appeared to be gradually remodeled over time, with blood vessels penetrating through it. However, some amounts of Norian SRS^®^ were detected in the medullary area as long as 78 weeks after being implanted in dog femurs [28]. An interesting study on the *in vitro* resorption of three apatite cements (conventional, fast-setting and anti-washout) by osteoclasts if compared with similar resorption of sintered HA and a cortical bone revealed an intermediate behavior of the cements: they were resorbed slower than bone but faster than HA [293]. Evidences of the direct contact of bone and a calcium orthophosphate cement without soft tissue interposition might be found in literature [294].

Different studies reported on both cement bioresorption and the progress of bone formation around calcium orthophosphate cements which in certain cases demonstrated both osteoconductive and osteoinductive properties [295]. However, some studies did not confirm the osteoinductive properties of calcium orthophosphate cements [296]. Some inflammatory reactions were noticed when the cement did not set [239]. As solubility of a non-stoichiometric CDHA is higher than that of stoichiometric HA, α- and β-TCP (Table 1) and the particle dimensions of precipitated CDHA is smaller than those of sintered calcium orthophosphates, the biodegradability of apatite cements is always better than that of dense bioceramics made of sintered stoichiometric calcium orthophosphates. For example, histologically, at two weeks, spicules of living bone with normal bone marrow and osteocytes in lacunae could be seen in the cement. At eight weeks, the cement was almost totally surrounded by mature bone. At this stage, no resorption of the cement was observed [297]. Only 30 % decrease of the implanted amount of Norian SRS^®^ was reported after 24 months in a rabbit femur [298]. Moreover, several differences can be expected depending on the cement type. For example, as the end-product of BoneSource^TM^ and Cementek^®^ is a very crystalline CDHA, BoneSource^TM^ and Cementek^®^ are expected to resorb slower than other apatite cements. Indeed no resorption of BoneSource^TM^ was observed after several years implantation; though some resorption of Biobone^®^ was detected. However, porosity appears to be the main biodegradability factor at play: a more porous (for cells) hardened cement degrades faster than a less porous one. For example, as Biobone^®^ is more porous than BoneSource^TM^, the discovered diversity could be due to the differences in the cement porosity [167,168]. The latter conclusion is confirmed by the results of other studies: a positive influence of the cement porosity on the resorption rate was found [237]. The interested readers are referred to the study on the suitability of porous calcium orthophosphate cements as scaffold material for bone regeneration, using a rabbit model [299].

The resorption properties of bioceramics are generally believed to relate to the solubility of their constitutive phases. The implanted calcium orthophosphates might be resorbed by two possible mechanisms, namely: an active resorption, mediated by the cellular activity of macrophages, osteoclasts and other types of living cells (so called phagocytosis or literally “cell-eating”) [300,301,302] and a passive resorption due to either chemical dissolution [11] or chemical hydrolysis (brushite cements only) [198] in the body fluids. Unfortunately, the factors concerning the biodegradation of calcium orthophosphate biomaterials have not been completely elucidated yet. The chemical composition, physical characteristics and crystal structures certainly play an important role in the biological behavior of calcium orthophosphates. In addition to this, the biodegradation may be influenced by the experimental conditions: experimental models, implantation sites and animal species [301].

Data are available indicating that macrophages and giant cells decompose quickly resorbed calcium orthophosphate cements (*e.g.*, brushite cements) [194], while slowly (from months to years) resorbed apatite cements are decomposed by osteoclast-type cells [26,178,303]. Clearly, a fast resorption of brushite cements can only be achieved if the cement resorption occurs before its conversion to CDHA according to equation (14) [41]. Both types of the resorption mechanisms (active + passive) might occur almost simultaneously, if a hardened cement consists of two different calcium orthophosphates, *e.g.,* from DCPD and β-TCP. For example, the biphasic brushite cement ChronOS™ Inject was found to resorb by dissolution with cement disintegration and particle formation followed by the phagocytosis of the cement particles through macrophages [304]. A similar cement was found to be degraded through a dissolution process associated with a cellular process. The observations suggested that cell activities could be influenced by a small particle size, without close correlation between the particle size and the cell activities but with a correlation between particle concentration and the cell activities [301]. The interested readers are referred to a very interesting review on the cellular mechanisms of calcium orthophosphate ceramic degradation [305].

A summary of brushite cement implantation studies in various animal models and defect locations is available in the literature [198]. Generally, in the same animal model, degradation rate decreases as sample size increases, as does DCPD to HA conversion time. The compositional changes of a brushite cement after implantation in sheep are described in detail elsewhere [285,306].

The kinetics of passive resorption depend on porosity of the samples, ionic substitutions, crystallinity and pH of the cement-tissue interface. The active resorption is due to cellular activity; however, it is also related to the passive one. Solution pH near macrophages and osteoclasts can drop to ~ 5 by the excretion of lactic acid, whereas near osteoblasts (bone forming cells) solution pH can become as high as 8.5 by the excretion of ammonia [25]. Dissolution chemistry of CDHA (therefore, of the hardened apatite cements) in acidic media (calcium orthophosphates are almost insoluble in alkaline solutions [10,11,189]) might be described as a sequence of four successive chemical equations [307,308]:

Ca_10-*x*_(HPO_4_)*_x_*(PO_4_)_6-*x*_(OH)_2-*x*_ + (2 – *x*)H^+^ → Ca_10-*x*_(HPO_4_)*_x_*(PO_4_)_6-*x*_(H_2_O)_2-*x*_^(2-*x*)+^ (15)

Ca_10-*x*_(HPO_4_)*_x_*(PO_4_)_6-*x*_(H_2_O)_2-*x*_^(2-*x*)+^ → 3Ca_3_(PO_4_)_2_ + (1 – *x*)Ca^2+^ + (2 – *x*)H_2_O (16)

Ca_3_(PO_4_)_2_ + 2H^+^ → Ca^2+^ + 2CaHPO_4_ (17)

CaHPO_4_ + H^+^ → Ca^2+^ + H_2_PO_4_^-^ (18)

Obviously, the dissolution chemistry of DCPD (therefore, of the hardened brushite cements) in acidic media is described by equation (18). One should stress that in equation (18) water is omitted for simplicity, therefore, dissolution of DCPA is written instead.

**Figure 4:**
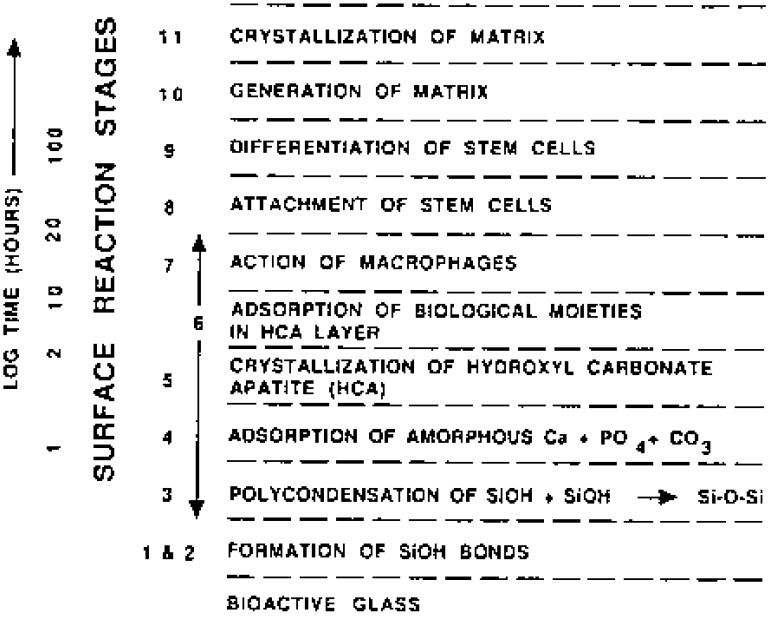
The sequence of interfacial reactions involved in forming a bond between tissue and bioactive glasses. The border between “dead” and “alive” occurs approximately at stage 6. For want of anything better, the bioactivity mechanism of calcium orthophosphate cements should also be described by this scheme with omitting of several initial stages, as it was made for HA in Ref. [315], where 3 initial chemical stages of the Hench’s mechanism were replaced by partial dissolution of HA. Reprinted from Ref. [314] with permission.

The mechanism of bone healing caused by calcium orthophosphate cements is very multifactorial because the surface of the cements is rapidly colonized by cells. Several types of these cells degrade calcium orthophosphates by either phagocytotic mechanisms (fibroblasts, osteoblasts, monocytes/macrophages) or an acidic mechanism with a proton pump to reduce the pH of the microenvironment and resorb the hardened bioceramics (osteoclasts) [305,309]. Various mesenchymal cells located at the implantation sites can induce solubilization of calcium orthophosphates. Upon the cells’ arrival, various active enzymes such as acid phosphatase are secreted, that cause dissolution of the hardened cements [310,311,312]. Much more biology, than chemistry and material science altogether, is involved into this very complex process and many specific details still remain unknown. Due to a lack of the necessary experimental data for calcium orthophosphates, the major bone healing steps caused by the cements might be schematically described by a modified scheme for the bioactivity mechanism of bioactive glasses – the concept introduced by Prof. Larry L. Hench [313,314]. The mechanism of bonding of bioactive glasses to living tissue involves a sequence of 11 successive reaction steps. The initial five steps occurred on the surface of bioactive glasses are “chemistry” only, while the remaining six steps belong to “biology” because the latter include colonization by osteoblasts, followed by proliferation and differentiation of the cells to form a new bone that had a mechanically strong bond to the implant surface (Figure 4).

It is well known that various polypeptides and growth factors present in bone matrix might be adsorbed onto HA and modulate the local milieu of cells. This is supported by many purification protocols of growth factors and bone morphogenetic proteins/osteogenins involving HA chromatography [316,317]. However, osteoblasts are not found in direct contact with calcium orthophosphates. A complex proteinaceous layer, usually osteoid, directly contacts the osteoblasts. After implantation of calcium orthophosphate cements, mitogenic events could occur either during the initial mesenchyma1 cell contact or after osteoid degradation by osteoblast collagenase. In a dense, mineralized material such as calcium orthophosphate cements, which provides a barrier to the free diffusion of circulating hormones, growth factors, and cytokines, it is questionable whether the local responses at the periphery of the material regulate osteoconduction [21]. The tissue response to injectable calcium orthophosphate cements is well described in literature [265,293,303,318,319]. Recent histological and mechanical evaluation of self-setting calcium orthophosphate cements in a sheep vertebral bone void model is available elsewhere [320]. The interested readers are also advised to get through a recent paper on the *in vitro* biodegradation of brushite cements by a macrophage cell-line [105].

To conclude this part, one should note that calcium orthophosphate cements are able to provide short-term biologically desirable properties and then be replaced by a new bone, which is very important [321]. The growth rate of a newly forming bone depends on age, sex and general metabolic health of the recipient as well as on the anatomic site, porosity, bulk site, crystallinity, chemical composition (brushite or apatite), particle sizes and P/L ratio of the cements. Considering all these factors, it might take from three to 36 months for different calcium orthophosphate cements to be completely resorbed and replaced by bone [172]. However, additional sound scientific data to determine the exact degree of biodegradability for calcium orthophosphate cements are still needed, viz. animal studies performed in a critical-size defect model. One must stress that the rate of cement resorption should be balanced with the rate of new bone formation to avoid collapse at the fracture site, which might occur if the resorption is too fast.

## 6. The Mechanical Properties

As in most clinical applications calcium orthophosphate cements are applied in direct contact with human trabecular bones, it may be stated as a mechanical requirement that the strength of the cements must be at least as high as that of trabecular bones, which is close to 10 MPa [322]. A three-dimensional (3D) complex load is applied during orthopedic and dental applications because of a combination of different forces that may include bending, torsion, tension and compression. Unfortunately, calcium orthophosphate cements are only strong enough under compression [165]. In theory, after setting, they can reach the mechanical properties comparable to those of calcium orthophosphate blocks with the same porosity. However, in practice, the strength of the cements is lower than that of bones, teeth or sintered calcium orthophosphate bioceramics [118].

Having a ceramic origin, the set products of all calcium orthophosphate cements are brittle, having both a low impact resistance and a low tensile strength (within 1 to 10 MPa), whereas the compression strength varies within 10 to 100 MPa [115,165,166]. The latter value exceeds the maximum compression strength of human trabecular bones. On the other hand, at 12 weeks after implantation the compressive strength of these cements was found to be still significantly higher (60 to 70 MPa) than that of normal bone [31]. Moreover, the mechanical properties of calcium orthophosphate cements are not narrowly distributed around a mean value (as for metals), but widespread over a very large range of values, which strongly reduces their clinical application [323]. Brushite cements are slightly weaker than apatite cements. A tensile strength of 10 MPa and a compressive strength of 60 MPa were obtained for brushite cements [324]. In comparison, apatite cements can reach a tensile strength of 16 MPa [325] and a compressive strength of 83 MPa [326]. *In vivo*, the difference between apatite and brushite cements boosts: namely, the mechanical properties of apatite cements were found to increase [291], whereas those of brushite cements decreased [31]. This is attributed to a higher solubility of DCPD when compared with that of CDHA (Table 1). After a few weeks of implantation, the mechanical properties of brushite cements began to increase due to bone ingrowth [31]. The interested readers are suggested to get through the mechanical characterization of a bone defect model filled with ceramic cements [170].

To improve the mechanical properties of calcium orthophosphate cements, addition of water-soluble polymers might be considered. For example, in early 1990s, Miyazaki *et al*. [327,328] used a number of polymers, including polyacrylic acid and polyvinyl alcohol to improve the properties of a TTCP + DCPD cement. They noted marked increases (up to threefold) in mechanical properties but with an unacceptable reduction of workability and setting time. Later, another research group reported similar results using sodium alginate and sodium polyacrylate [329]. Afterwards, other researchers added several polyelectrolytes, polyethylene oxide and a protein bovine serum albumin into α-BSM™ cement paste to create calcium orthophosphate – polymer composites [330]. Composites of α-BSM™ with polycations (polyethylenimine and polyallylamine hydrochloride) exhibited compressive strengths up to six times greater than that of pure α-BSM™ material. Composites of α-BSM™ with bovine serum albumin developed compressive strengths twice that of the original α-BSM™ cement [330]. Similar strengthening effect was achieved by addition of some commercial superplasticizers [331]. The results showed that small additions, *i.e*. 0.5 vol. %, in the aqueous liquid phase improved the maximum compressive strength (35 MPa) of Biocement-H^©^ by 71 %, *i.e*. till 60 MPa. Moreover, the addition of high amounts of superplasticizers, *i.e*. 50 vol. %, allowed for a significant increasing of the P/L ratio from 3.13 to 3.91 g/ml, without affecting the maximum strength and/or the workability of the cement [331]. This effect was explained by an inhibiting effect of the aforementioned additives on the crystal growth kinetics of newly forming crystals of calcium orthophosphates, which resulted in smaller crystallites and, hence, a denser and more interdigitated microstructure. However, the increased strength was attributed mainly to the polymer’s capacity to bridge between multiple crystallites (thus forming a more cohesive composite) and to absorb energy through a plastic flow [330].

As the presence of pores makes it easier for cracks to run throughout the hardened mass, the mechanical properties of the hardened cements were found to decrease exponentially with a porosity increase [332]. In theory, calcium orthophosphate cements can be made with almost any porosity. However, for most commercial cements, the pores are of 8 – 12 μm in diameter and, after the cement is set, about 40 – 50 % of its volume is the porosity [333]. Pressure can be applied to reduce the porosity of calcium orthophosphate cements [118,334,335]. The pore dimensions in hardened cements are too small to allow fast bone ingrowth. There is a lack of macroporosity. Besides, unless the special efforts have been performed, the available macroscopic pores are not interconnected. Due to these reasons, after injection, osteoclastic cells are able to degrade the hardened cements layer-by-layer only, starting at the bone-cement interface throughout its inner part (in other words, from the outside to the inside). This is the main drawback of the classical cement formulations when compared to calcium orthophosphate ceramic scaffolds with an open macroporosity [167,168].

Since the compression strength is reciprocally proportional to porosity, the former might be adjusted by varying the P/L ratio in the hardening mixture. Elevated compression strength would be applicable in cranioplasty for regions requiring significant soft-tissue support. For smaller bone defects, such as root canal fillings, low-compression cements might be used [111]. Concerning the tensile strength of calcium orthophosphate cements, as a rule of thumb, it appears to increase two-fold with each 10 vol. % decrease of the porosity, *i.e.* 5, 10, 20, 40 and 80 MPa for 80, 70, 60, 50 and 40 % porosity, respectively [167,168]. The effect of porosity on the compressive modulus of calcium orthophosphate cements is available as Figure 4 in Ref. [335]. Ishikawa and Asaoka showed a linear relation (R^2^ = 0.94) between ln diametral tensile strength and porosity of a calcium orthophosphate cement where porosity was controlled by compaction pressure (up to 173 MPa) [115]. Besides, an empirical relationship between strength, S, and porosity, P is also available [336]:

S = S_0_e^−*b*P^,

where S_0_ is the theoretical strength at P = 0 (fully dense) and *b* is an empirical constant.

As the porosity is mainly due to an excess of water used in the cement compositions, attempts were made to reduce the amount of water. Besides, the amount of water determines the rheological properties of the cement paste: a decrease in water content leads to a large increase in viscosity, eventually leading to non-flowable pastes. As calcium orthophosphate cements set at an almost constant volume, the final porosity can be predicted from the initial composition [167,168]. A shrinkage degree of ~ 1 % causes no restrictions on clinical use [163]. Recent studies on the *in vivo* evaluation of an injectable macroporous calcium orthophosphate cements revealed a higher bioresorption rate due to both a higher surface contact with body fluids (which increases dissolution) and enhancing cellular activity due to particle degradation [237,265].

According to Bohner [167], it is difficult to compare the mechanical properties of different cement formulations. For example, the following numeric values of the compression strength and setting time were obtained for Norian SRS^®^: 33 ± 5 MPa and 8.5 ± 0.5 min (≈ 50 % porosity), Cementek^®^: 8 ± 2 MPa and 17 ± 1 min, Biocement D^®^: 83 ± 4 MPa and 6.5 ± 0.5 min (≈ 40 % porosity) and α-BSM^®^: 4 ± 1 MPa and 19 ± 1 min (≈ 80 % porosity), respectively [326]. Among them, Biocement D^®^ has the highest compressive strength but the lowest porosity. A high compressive strength does not necessarily mean that Biocement D^®^ is the least breakable implant. *In vivo*, shear and tensile forces indeed play a very important role. Therefore, the tensile strength of the cements should also be considered, for example, using the Mohr circle approach [337]. Finally, it should be kept in mind that the initial mechanical properties of calcium orthophosphate cements may vary with implantation time. Animal studies indicated that the mechanical properties of apatite cements tended to increase continually [291], in contrast to those of brushite cements, which initially decreased and again increased when bone was growing [31]. Further details on the major properties of Norian SRS^®^ are available elsewhere [173,338].

The porosity level of calcium orthophosphate cements might be controlled to a certain extent by adjusting particle sizes and the P/L ratio. When the P/L ratio is high, the porosity of the apatite cement is low [167,168]. Besides, successful attempts have been made to introduce macroporosity into calcium orthophosphate cements by using soluble particles (porogens) [248,251,274,336,339], resorbable polymers [340,341], fast resorptive phases [188,249] or foaming agents (*e.g.,* dehydrated albumen) [237,249]. According to calculations, the tensile strength of the cements with zero porosity could be as high as 103 MPa [115]. However, a high density and a lack of pores decreases cement bioresorbability because a newly forming bone appears to be unable to grow into the pores; it might grow only simultaneously with dissolution of the cement. Thus, the porosity of calcium orthophosphate cements is a very important factor for the cement degradability [167,168]. Other factors affecting strength are the materials used in the solid phase, particle sizes, incorporation of fillers into the solid phase, the P/L ratio and various liquid phases [93]. The strength of the cement-prosthesis interface might be studied by a pullout test. The details are available elsewhere [57].

## 7. Reinforced Calcium Orthophosphate Cement Composites and Concretes

Being aware of the excellent bioresorbability of DCPD and CDHA, researchers are focused on attempts to overcome the mechanical weakness of calcium orthophosphate cements by using different fillers, fibers and reinforcing additives that give rise to formation of various multiphasic composites [91,92,96,169,226,238,333,336,342,343,344,345,346,347]. Even carbon nanotubes have been successfully tested to reinforce calcium orthophosphate cements [348]. Although the biomaterials community does not use this term, a substantial amount of such formulations might be defined as calcium orthophosphate concretes [349]. The idea behind the concretes is simple: if a strong filler is present in the matrix, it might stop crack propagation. However, adding fillers always reduced the porosity that negatively influenced the ability of the concretes to allow bone ingrowth into pores. Hence, a denser cement has a slower resorption rate and thus a slower bone substitution [115]. Moreover, due to the presence of fillers, the rheological properties and injectability of calcium orthophosphate concretes frequently appear to be worse than those properties of calcium orthophosphate cements. Thus, it is difficult to increase strength of the cements without having a negative influence on the other properties.

Calcium orthophosphate concretes can be prepared from both apatite and brushite cement formulations. For example, in an attempt to improve the mechanical properties of calcium orthophosphate cements, a group of investigators prepared concretes by adding human cadaveric femur bone chips in amounts of 25, 50 and 75 % (w/w) to α-BSM^®^ cement [343]. The mechanical tests revealed that the specimens of pure cement exhibited a relatively high stiffness but a low ductility. However, for the cement-bone concretes an increase of bone content was found to result in the elastic modulus decreasing and the ductility increasing; however, the ultimate strength showed only small changes with no apparent trend [343]. A concrete of Biopex^®^ cement with allografts taken from femurs and tibiae of rabbits is also available. Unfortunately, nothing is written on the mechanical properties improvement but, surprisingly, by the addition of allografts, the hydrolysis process of Biopex^®^ was significantly changed [226]. By adding polymers and composites, other researchers succeeded in improving the mechanical strength of the cements up to 30 MPa; however, the kinetics of CDHA formation and thus the bioactivity of the material were decreased [97,350]. Xu *et al.* reported that incorporation of long carbon fibers at a volume fraction of 5.7 % increased the flexural strength about four-fold and fracture work 100-fold, if compared to un-reinforced calcium orthophosphate cements [351]. The reinforcement mechanisms were found to be crack bridging and fiber pullout, while fiber length and volume fraction were key microstructural parameters that determined the concrete properties [351]. Although addition of polypropylene, nylon and carbon fibers was found to reduce the compression strength of a double-setting calcium orthophosphate cement because of increased porosity, it strongly increased the cement’s fracture toughness and tensile strength, relative to the values for the un-reinforced variant of this cement [344]. A knitted two-dimensionally oriented polyglactin fiber-mesh was found to be effective in improving load-bearing behavior of a calcium orthophosphate cement for potential structural repair of bone defects [169]. To make the material stronger, fast setting and anti-washout, chitosan was added to the cements [280,327,352,353,354,355,356,357,358,359,360,361]. Calcium orthophosphate cements doped by SiO_2_, and TiO_2_ particles showed a significant (~ 80 – 100 MPa) increase in the compressive strength, whilst no change in the mechanical behavior of the cements was observed when ZrO_2_ particles were added [345]. Besides, calcium orthophosphate cements might be successfully reinforced by addition of calcium silicates [59], polypeptide copolymers [362] and collagen [363,364,365].

Yet another team examined the effects of varying fiber type, fiber length and volume fraction of fiber-reinforced calcium orthophosphate concretes [352,366]. Four fiber types were studied: aramid, carbon, E-glass and polyglactin. Fiber length ranged from 3 – 200 mm and fiber volume fraction ranged from 1.9 – 9.5 %. The results indicated that a self-setting calcium orthophosphate cement was substantially strengthened via fiber reinforcement. Aramid contributed to the largest increase in composite strength, followed by carbon, E-glass and polyglactin. Fiber length, fiber volume fraction and fiber strength were found to be key microstructural parameters that controlled the mechanical properties of calcium orthophosphate concretes [352,366]. Fiber reinforcement of porous cements (mannitol was used as a porogen) was discovered as well [367]. Namely, reinforcement by aramid fibers (volume fraction of 6 %) was found to improve the properties of a calcium orthophosphate cement with the strength increasing threefold at 0 % mannitol, sevenfold at 30 % mannitol and nearly fourfold at 40 % mannitol. Simultaneously, the work of fracture increased by nearly 200 times, however the modulus was not changed as a result of fiber reinforcement [367]. Addition of 20 wt. % of acrylamide and 1 wt. % ammonium polyacrylate to the liquid increased the compressive and tensile strength of α-TCP bone cement by 149 and 69 % (55 and 21 MPa), respectively [368]. A positive influence of polyamide fibers [369] and bioactive glass [370] is also known.

In the cases when bioresorbable reinforcement fibers are used, strength augmentation is attained at the initial stages [340,371,372,373,374]. For example, the initial strength of a concrete was threefold higher than that of the unreinforced cement control [371]. The work of fracture (toughness) was found to increase by two orders of magnitude for other composites of calcium orthophosphate/resorbable fiber (namely, Vicryl polyglactin 910, Ethicon, Somerville, NJ, USA [372] and a mesh of copolymer of polyglycolic and polylactic acids [340]). When implanted *in vivo*, bioresorbable fibers would provide initial strength and then dissolve to form interconnecting macroscopic channels, which could facilitate bone ingrowth into the implant [117,118,340,371]. For example, interconnected macropores were formed in a calcium orthophosphate cement at 84 days’ immersion in a physiological solution [340]. One should note that, apart from the mechanical properties of the reinforcing material, the structure of the incorporated fibers, regular or random, appears to be crucial for the resulting flexural strength and modulus of elasticity [374]. A higher strength might help extending the use of calcium orthophosphate cements to larger stress-bearing repairs, while the macropores might facilitate tissue ingrowth and integration of the cement with an adjacent bone. To extend this idea further, several types of fibers with different rates of bioresorbability might be simultaneously incorporated into a cement formulation.

Besides the aforementioned, it is important to mention concretes, after hardening consisting of calcium orthophosphates only [205,304,375,376,377,378]. The first biphasic composition consisting of a hardened DCPD matrix filled with β-TCP granules was introduced in 1992 [376]. Further development of this formulation might be found in other papers [205,304]; unfortunately, neither the mechanical nor the rheological properties of this concrete have been disclosed. At physiologic pH, the *in vitro* solubility of DCPD is approximately 100 times higher than β-TCP; roughly, the same order of magnitude applies for the *in vivo* resorption kinetics of these calcium orthophosphates. A new bone forms in the space left after resorption of the DCPD matrix, while β-TCP granules act as guiding structures. This feature of the cement can be considered an inverse scaffolding effect [379]. Another group of investigators invented a formulation that incorporated as major powder components α-TCP, ACP and biphasic calcium phosphate (BCP; consisting of an intimate mixture of HA and β-TCP in various HA/β-TCP ratios) [342]. It was believed that after setting such a formulation could provide a porous ceramics *in vivo* due to preferential dissolution of a better soluble ACP component compared to the other calcium orthophosphates in the matrix. Further, this combination was extended to a multiphase concrete composition consisting of 70 % w/w settable matrix (mixture of 45 % α-TCP, 5 % MCPM and 25 % ACP [380]) with the average particle dimensions of 15 µm and 30 % BCP granules (ranging between 80 and 200 µm) as a filler [375]. The role of BCP granules is quite interesting: after implantation of a cement without BCP granules, the quality of newly formed bone was not identical to the host bone, while implantation of a concrete with BCP granules resulted in formation of a new bone identical to the host bone. The reason of this phenomenon is not clear yet; but, perhaps, it correlates with similar results for β-TCP granules, which act as bone anchors and encourage formation of a mature bone [205,206].

Effects of added α-TCP and β-TCP were investigated to shed light on the setting reaction of apatite cement consisting of TTCP and DCPA [378]. Added β-TCP showed no reactivity, and thus resulted in extended setting time and decreased mechanical strength. In contrast, α-TCP dissolved to supply calcium and orthophosphate ions after initial apatite crystal formation by the chemical reaction (1). Although setting time was delayed because α-TCP was involved only in the latter reaction of apatite cement, larger apatite crystals were formed due to its addition. Because of larger apatite crystal formation, the mechanical strength of α-TCP-added apatite cement increased by approximately 30 %, as compared to α-TCP-free apatite cement [378].

 A strength improvement was found when DCPA and TiO_2_ crystals were used as fillers for mechanically activated α-TCP cements [381]. Calcium orthophosphate concretes reinforced by whiskers made of calcium carbonate [47] and HA [377] have been also developed.

To conclude this part, one should briefly mention on the reverse situation: there are bone concretes made of acrylic cements, reinforced by calcium orthophosphate powders or granules [382,383,384,385,386,387]. The calcium orthophosphates presented in these formulations act as fillers, which are necessary to improve the mechanical properties and to impart bioactivity; they do not participate in the hardening mechanisms. Polymerization of monomers is primarily responsible for setting of such composites and concretes. However, that is another story.

## 8. Clinical and Medical Applications

Injectable osteoconductive calcium orthophosphate cements have been introduced as an adjunct to internal fixation for treating selected fractures. Different studies have already shown that they are highly biocompatible and osteoconductive materials, which can stimulate tissue regeneration [21,389]. The main purpose of calcium orthophosphate cements is to fill voids in metaphyseal bone, thereby reducing the need for bone graft, although the cements also might improve the holding strength around metal devices in osteoporotic bone. Bone augmentation (*i.e*., a reinforcement of osteoporotic bone through injection) appears to be a very promising application field of calcium orthophosphate cements. Such procedures ease the fixation of screws in mechanically poor bone (for example for osteosynthesis) and decrease the pain associated with unstable vertebrae. The combination of a self-setting nature, moldability, biocompatibility, lack of any by-products and a great potential for being replaced by bone make calcium orthophosphate cements very promising materials for clinical applications: they can easily be used by bone remodeling cells for reconstruction of damaged parts of bones [89,90,194,319,390,391]. The ability to be molded in place also is a very important property, because a cement can easily be delivered into the desired place and can be fitted perfectly with bone defects [90]. Besides, some formulations were found to possess an antimicrobial activity [48,51,53,60,392], as well as promote osteoblast cell adhesion and gene expression *in vitro* [393].

Recent studies reported optimistic results in relation to the clinical application of calcium orthophosphate cements. For example, the data on cytocompatibility and early osteogenic characteristics are available in literature [394]. The ratio of the cases determined to be “effective” or “better” among the 74 cases we found to be 97.3 % [395]. Besides, the results of intra-articular degradation and resorption kinetics of these cements revealed no signs of pronounced acute or chronic inflammation [396]. Injected Norian SRS^®^ cement was mainly found as a single particle, anterior to the cruciate ligaments. The cement became surrounded by synovial tissues within four weeks and showed signs of superficial resorption [396]. Unfortunately, disintegration or washout of calcium orthophosphate cements has been reported as a potential clinical problem [115,179]. Perhaps, this problem could be solved by putting pressure on the paste during the setting period. In addition, sodium alginate might be added; however, the mechanical properties (strength) of this formulation are still poor [95].

According to the available information, the first animal study of calcium orthophosphate cements was performed in 1987 [126]. Afterwards, in 1991, a cement consisting of TTCP and DCPA was investigated histologically by implanting disks made of this cement within the heads of nine cats [397]. Simultaneously, another research group evaluated the tissue reaction to this cement in the teeth of monkeys [398]. The important examples of the most significant directions of current medical applications of calcium orthophosphate cements and concretes are given below.

### 8.1. Dental Applications

A group of investigators extracted all mandibular premolar teeth from beagles [399]. After one month of healing, alveolar bone was reduced to make space for a previously fabricated calcium orthophosphate cement block. One more month later, 8-mm HA implants were placed in such a manner that the apical half was embedded into alveolar bone and the coronal half in the calcium orthophosphate cement block. The investigators observed that the cement block was gradually replaced by bone and histopathologic features of the cement area were similar to that of natural bone. Moreover, the coronal half of the implants, previously surrounded by the calcium orthophosphate cement, was firmly attached by natural bone [399]. In another study, the same researchers used fluorescent labeling analysis and electron microanalysis to measure the extent of new bone formation and elemental (Ca, P, Mg) distribution [400]. The results indicated the presence of newly formed bone at one month after surgery and similar elemental distributions in the calcium orthophosphate cement and natural bone areas at six months after surgery [172]. Besides, calcium orthophosphate cements were tried as root canal fillers [51,401,402] and for pulp capping [403].

A hydraulic calcium orthophosphate cement was injected as a bone filler for gaps around oral implants placed on the medial femoral condyles of six goats and found excellent bone formation around the graft material. Unfortunately, the degradation rate of the cement appeared to be very slow and no resorption was observed [404]. In another study, a cement was placed on artificially created periodontal defects but no significant difference was found between the cement and control. However, the cement acted as a scaffold for bone formation and provided histocompatible healing of periodontal tissues [405]. Still other investigators used a cement for direct pulp capping and compared it to calcium hydroxide. Both materials were found to be equally capable of producing a secondary dentin at 24 weeks [406].

### 8.2. Craniofacial and Maxillofacial Applications

The use of calcium orthophosphate cements for craniofacial applications seems logical, as there is little or no stress generated under these conditions. Moreover, the ability to mold the material at placement is an enormous advantage from a cosmetics standpoint [172]. For example, BoneSource^TM^ is indicated for the repair of neurosurgical burr holes, contiguous craniotomy cuts and other cranial defects with a surface area no larger than 25 cm^2^ per a defect. In addition, it may be used in the sinus region for facial augmentation [90,407] and the cement can be supported by metal hardware [90]. In dogs, BoneSource^TM^ was employed to supplement the supraorbital ridge and to augment skull base defects [408]. Another group performed trials to ascertain the inflammation around the site and the degree of loss of the implanted BoneSource^TM^. The material was found to be osteoconductive with both periosteal and endosteal bone formation [409]. One more group presented excellent results using the material combined with an underlying resorbable mesh in calvarian defects of Yorkshire pigs. They found progressive bone ingrowths in all defects at 180 days, with nearly complete replacement by host bone [341]. Besides, excellent results for over 100 human patients were reported when a calcium orthophosphate cement was used in cranial defects. The success rate of the cement after six years was 97 % [81]. The results of still other medical trials are available elsewhere [189,410,411,412,413,414,415,416,417].

### 8.3. Orthopedic Applications

Calcium orthophosphate cements have successfully been used for treatment of distal radius fractures [175,418,419]. Besides, other successful attempts have been made to use the cements for calcaneal fractures [420], hip fractures [421,422], augmentation of osteoporotic vertebral bodies [423], tibial plateau fractures [29,424,425,426,427], restoration of pedicle screw fixation [428], reinforcement of both thoracolumbar burst fractures [429], cancellous bone screws [430], in wrist arthrodesis [431] and for fixation of titanium implants [432]. A recent study on a cement augmentation of the femoral neck defect might be found elsewhere [433]. Considering their properties, calcium orthophosphate cements might potentially be applied to reinforce osteoporotic vertebral bodies [423,434]. Further details are available elsewhere [435,436]. Besides, calcium orthophosphate cements appear to be a reliable subchondral replacement material when the bone defect is adjacent to the articular cartilage [437].

### 8.4. Vertebroplasty and Kyphoplasty

Vertebroplasty and kyphoplasty are two surgical procedures that recently have been introduced to medically manage of osteoporosis-induced vertebral compression fractures. Particularly, both procedures aim to augment the weakened vertebral body, stabilize it and/or restore it to as much of its normal height and functional state as possible. Both procedures involve injection of a self-setting paste of a calcium orthophosphate cement into the fractured vertebral body, which resulted in a faster healing [81,173,437,438,439,440,441,442,443]. Furthermore, prophylactic injections of calcium orthophosphate cements also have been performed.

### 8.5. Drug Delivery

In general, a potential substrate to be used as a drug carrier must have the ability to incorporate a drug, retain it in a specific target site and deliver it progressively with time in the surrounding tissues. Additional advantages are provided if the material is injectable, biodegradable, sets at ambient temperature, has near neutral pHs and a large surface area [32,33]. These properties make calcium orthophosphate cements to be very attractive candidates as drug carriers for therapeutic peptides [445], antibiotics [446,447,448,449,450,451,452,453,454,455], anticancer drugs [456], anti-inflammatory drugs [457,458], cytokines [459], hormones [460] and bone morphogenetic proteins [359,461,462,463,464,465]. For example, a “growth factor cement (GFC)” has been reported [466]. In that study, a combination of bone morphogenetic protein-2 (BMP-2), transforming growth factor-beta (TGF-β1), platelet-derived growth factor and basic fibroblast growth factor (bFGF) was used in a calcium orthophosphate cement for treatment of peri-implant defects in a dog model. The findings indicated a significant effect of GFC on increased bone-to-implant contact and amount of bone per surface area if compared with both the cement-only and no-cement treatment groups [466]. Similar data were found for a combination of a calcium orthophosphate cement with an exogenous nerve growth factor [467]. Even more complicated combination of deproteinized osteoarticular allografts integrated with a calcium orthophosphate cement and recombinant human vascular endothelial cell growth factor plus recombinant human BMP-2 (rhBMP-2) has been studied as well [468].

In principle, drugs might be incorporated into both a liquid and a powder phase of the cements. After setting, the drugs are slowly released through the cement pores [179,451,452,453,454,469,470]. For example, a group of investigators added flomoxef sodium to a cement formulation and found that the release of the antibiotic could be easily controlled *in vivo* by adjusting the content of sodium alginate in the formula [179]. *In vitro* elution of vancomycin from calcium orthophosphate cement has been studied as well [470]. The possibility of using calcium orthophosphate cements as a drug-delivery system offers an attractive and efficient solution for the treatment of various bone diseases, *e.g.,* tumours, osteoporosis and osteomyelitis, which normally require long and painful therapies.

The laboratory studies on drugs incorporation into the cements cover different aspects. Firstly, it is necessary to verify that addition of a drug does not influence the setting reaction not only in terms of the setting and hardening mechanisms but also with respect to the rheological behavior and injectability. Secondly, it is necessary to determine the *in vitro* kinetics of drug release. Thirdly, the drug delivery properties of the cement must be studied *in vivo*. Finally, but still importantly, the clinical performance of the drug delivery system must be evaluated as well [32,33]. For example, recombinant human transforming growth factor β1 (rhTGF-β1) was added to a calcium orthophosphate cement [471,472,473,474]. This resulted in formation of a bioactivated cement that could be used as a bone filler and for the replacement of bone [471]. It appeared that after 8 weeks the addition of growth factors stimulated and increased bone formation (50 % volume) and bone contact (65 %) in comparison to control calvarian defects in an animal study. Besides, the growth factor group reduced the remaining volume of the cement by 20 % [472]. Examples of rhBMP-2 release from a loaded porous calcium orthophosphate cement might be found elsewhere [474,475], while an experimental study on calcium orthophosphate cement impregnated with dideoxy-kanamycin B is also available [476].

Although most materials currently used as drug carriers are polymers, in the specific field of the pharmacological treatment of skeletal disorders, calcium orthophosphate cements have an added value due to their bioactive character and injectability. Further details and additional examples of the drug delivery application of calcium orthophosphate cements are well described elsewhere [25,32,33].

### 8.6. Brief Conclusions on the Medical Applications

To conclude this part, one should stress that despite several encouraging results, not every surgeons’ expectations have been met yet [476]. First of all, calcium orthophosphate cements and concretes are not superior to autografts, despite offering primary stability against compressive loading [477,478]. One of the main concerns of clinicians is to reach higher rates of bioresorption, an improvement of bone reconstruction and to a lesser extent, higher mechanical resistance [29]. Besides, clinical application of the cements in comminuted fractures revealed penetration of the viscous paste into the joint space [479,480,481]. The interested readers are referred to a paper on cement leakage during vertebroplasty [482]. To date, cadaver studies have already shown that using calcium orthophosphate cements with conventional metal fixation in certain fractures of the distal radius, tibial plateau, proximal femur and calcaneus can produce better stability, stiffness and strength than metal fixation alone. Early clinical results have revealed a reduced time to full load bearing when the cements were used for augmentation of tibial plateau and calcaneal fractures, more rapid gain of strength and range of motion when used in distal radius fractures and improved stability in certain hip fractures [391,418]. However, surgeons reported on difficulties in filling the vertebral bodies (a bad injectability of present formulations) and other problems, such as filter-pressing and cement decohesion, observed during vertebral body injection that resulted in bone instability due to low mechanical strength as well as long setting times of the cements [483]. This happens due to not only low mechanical properties of calcium orthophosphate cements but also some difficulties of filling vertebral bodies. In order to maintain a good cohesion and reduce filter-pressing, calcium orthophosphate cements need to be more viscous (hence, less injectable) [167,168]. For example, calcium orthophosphate cements might be modified by addition of polysaccharides [84,95,271,272,273,274] and/or gelatin [240,275,276,277,278,279,280].

Another type of concerns that has been raised is that the use of calcium orthophosphate cements for the augmentation of fractured and osteoporotic bones might aggravate cardiovascular deterioration in the event of pulmonary cement embolism by stimulating coagulation [484]. To investigate these potential problems, 2.0 mL of either calcium orthophosphate or polymethylmethacrylate (PMMA) cement were injected intravenously in 14 sheep. Intravenous injection of calcium orthophosphate cement resulted in a more severe increase in pulmonary arterial pressure and decrease in arterial blood pressure compared to the PMMA cement. Disintegration of the calcium orthophosphate cement seemed to be the reason for more severe reaction that represents a risk of cardiovascular complications. The authors concluded that further research efforts should aim at improving cohesion of calcium orthophosphate cements in an aqueous environment for future clinical applications such as vertebral body augmentation [484].

To conclude the medical part of this review, one should mention that, although the long-term outcomes are still poorly documented, currently there are no doubts concerning a very great potential of the clinical applications of calcium orthophosphate cements and concretes for healing of bone and dental defects. For example, a bioresorbable calcium orthophosphate cement was once found to be a better choice, at least in terms of the prevention of subsidence, than autogenous iliac bone graft for the treatment of subarticular defects associated with unstable tibial plateau fractures [485]. Furthermore, BoneSource^TM^ was found to be safe and effective when used to fill traumatic metaphyseal bone voids and appeared to be at least as good as autograft for treatment of these defects [486]. As this manuscript is intended to be read mainly by chemists and materials researchers, the biological, medical and clinical aspects of calcium orthophosphate cement applications have not been discussed in many details. For further biomedical details, the interested readers are referred to other papers and reviews [21,25,32,33,111,391,395,477].

## 9. Future Developments

As calcium orthophosphate cements and concretes represent an intriguing group of new materials for bone augmentation and reconstruction, there is a great potential for further improvement of their properties, in which the ideal characteristics (Table 4) should be approached by manipulations of the chemical composition, powder particle size and distribution, as well as by means of various additives. Several commercial cement formulations have been already approved for a clinical application [111,175,397,410]. New formulations of both apatite and brushite cements are expected to appear in the market soon. The forthcoming commercial formulations will need to be improved in order to take the advantage of a variety of possibilities offered by calcium orthophosphate cements. New formulations will include (i) injectable and open macroporous formulations to optimize their osteoconduction [240], (ii) formulations containing only one calcium orthophosphate (single-phase cement powders) [17] and (iii) drug-loaded and hormone-loaded cements for the treatment of bone diseases [25,32,33]. Obviously, the former two directions deal with both chemistry and material science, while the last direction is more related to tissue engineering and medicine.

Two innovative approaches of injectable cement formulations have been introduced relatively recently. The researches combined a water-reactive apatite cement such as a mixture of TTCP and DCPD powders with a nonaqueous but water-miscible liquid (*e.g.,* glycerol, polyethylene glycol) + a gelling agent (*e.g.*, hydroxypropylmethylcellulose, carboxymethylcellulose, chitosan) + a hardening accelerator (*e.g.*, tartaric acid, malic acid, malonic acid, citric acid or glycolic acid) to form a stable paste that can be directly injected into a bone defect [487,488,489]. In literature, this type of cement pastes is called “premixed calcium phosphate cements” (occasionally referred to as PCPC) in which the paste remains stable during storage and hardens only after placement into the defect. Setting occurs upon contact with body fluids or in a physiological solution and results in CDHA formation. This approach eliminates the powder-liquid mixing stage during surgery and might improve the cement performance. Besides, it allows shortening the surgical time, as well as the risk of operator-induced error is considerably reduced.

The first formulation of premixed calcium orthophosphate cements had a setting time of longer than 1 h and a low mechanical strength [487]. Afterwards, an improved formulation has been developed; it exhibits a rapid setting when immersed in a physiological solution, yielding a hardened cement with a higher mechanical strength, approached the reported strengths of sintered porous HA implants and cancellous bone [488,489]. Creation of premixed macroporous calcium orthophosphate cement scaffolds reinforced by slow-dissolving fibers (in other words, premixed macroporous concrete scaffolds) is the latest achievement of this approach [339]. Other researchers invented cements in the form of two injectable pastes that could be mixed together and injected at the time of implantation (with a static mixer incorporated in the injection cannula) [490]. Nevertheless, the latter approach is limited to acid-base cement formulations only [30].

**Table 4:** Major advantages and disadvantages of the calcium orthophosphate cements [32,33,172].

Advantages	Disadvantages
Self-setting ability *in vivo*. Good injectability that allows cement implantation by minimally invasive surgical techniques, which are less damageable than the traditional surgical techniques. Good osteoconductivity and occasional osteoinductivity: the initial biological properties of the hardened cements are similar to those of CDHA or brushite. Can be replaced by newly formed bone after a period of time (osteotransductivity). Moldability: the perfect fit to the implant site, which assures good bone-material contact, even in geometrically complex defects. Excellent biocompatibility and bioactivity. No toxicity. Low cost. Ease of preparation and handling. Setting at body temperature. Form chemical bonds to the host bone. Clinically safe materials in their powder components. Can be used to deliver antibiotics, anti-inflammatory drugs, growth factors, morphogenic proteins, *etc*. at local sites, which are able to stimulate certain biological responses.^*^	Mechanical weakness: limited use due to potential collapse of material followed by soft tissue formation instead of bone formation (loaded areas). Until cements with adequate shear strength are available, most complex fractures that can be repaired with cement also will require metal supports. Can be washed out from surgical defect if excess of blood. Lack of macroporosity (especially interconnected pores), which prevents fast bone ingrowth and the cements degrade layer-by-layer from the outside to the inside only. The *in vivo* biodegradation of many formulations is slower than the growth rate of a newly forming bone.

^*^ Further studies are necessary.

To date, no study has reported on a possibility of the premixed brushite formulations at ambient temperatures. However, recently the researchers have discovered a way to overcome this problem at low temperatures [129]. Three different pre-mixed brushite cement formulations formed by freezing the cement pastes following combination of the powder and liquid components. When frozen and stored at – 80 °C or less, significant degradation in compression strength did not occur for the duration of the study (28 days). Interestingly, in the case of the brushite cement formed from the combination of β-TCP with 2 M H_3_PO_4_ solution, freezing the cement paste had the effect of increasing mean compressive strength fivefold (from 4 to 20 MPa), which was accompanied by a reduction in the setting rate of the cement. This strength improvement was attributed to a modification of the crystal morphology and a reduction in damage caused to the cement matrix during manipulation [129].

A lack of macropores is a substantial disadvantage of many current formulations of calcium orthophosphate cements [237]. As a result, biodegradation takes place layer-by-layer on the surface, from outside to inside. To solve this problem, soluble particles, such as sugar [491], mannitol [336,340,492], NaCl [258,259] and calcite [188], or resorbable fibers [340,366,367,368] might be incorporated into the cement. After the cement is implanted, the particles are dissolved, leaving pores in the cement matrix; however, such pores are not always interconnected. Using a hydrophobic liquid instead of soluble particles could be an alternative. At the turn of the millennium, an open macroporous structure was obtained using a mixture of oil and a cement paste [493]; however, since than no research papers on this subject have been published. Besides, by means of surfactants, air bubbles might be created in the bulk of the cements [269]. Finally, addition of carbonates to the cement formulation is able to create pores [29,46,264,265]. Unfortunately, the mechanical strength and porosity are conflicting requirements. As the porosity in calcium orthophosphate cements appears to be of paramount importance to achieve the excellent bioresorbability, other experimental approaches have to be developed [494].

Recently, a layered structure was designed by combining a macroporous layer of calcium orthophosphate cement with a strong fiber-reinforced calcium orthophosphate cement layer. The rationale for such construction was for the macroporous layer to accept tissue ingrowth, while the fiber-reinforced strong layer would provide the needed early-strength [495].

In the case of calcium orthophosphate concretes, future studies could combine in one formulation porogens and biodegradable fibers of different shapes and dissolution rates to form after *in vivo* hardening calcium orthophosphate scaffolds with sustained strength. In such a system, one porogen quickly dissolves and creates macropores to start a bone ingrowth process, while the second type of fibers provides the required strength to the implant. After significant bone ingrowth into the initial pores increased the implant strength, the second set of fibers would then dissolve to create additional macropores for bone ingrowth [371]. Such complicated formulations have already been developed. For example, chitosan, sodium orthophosphate and hydroxypropylmethylcellulose were used to render calcium orthophosphate cement fast setting and resistant to washout, while absorbable fibers and mannitol porogen were incorporated for strength and macropores, respectively. Both the strength and fracture resistance of this concrete were substantially increased and approached those values for sintered porous HA implants [496]. Turning on a bit of imagination, one might predict development of polymeric drugs [497], hormones, growth factors, *etc*. (*e.g.*, by either incorporation into or cross-linking with either water-soluble or bioresorbable polymers). Coupled with reinforcing biodegradable fibers and porogens, such types of “healing fibers” might be added to calcium orthophosphate concretes, which not only accelerate the remedial process, but also allow simultaneous improvement in both their strength and injectability.

Stability (insolubility) in normal physiological fluid environment and resorbability under acidic conditions produced by osteoclasts appears to be among the most important *in vivo* characteristics of modern calcium orthophosphate cements and concretes. For some clinical applications, such as cranioplasty, a relatively slow resorption and replacement by bone is quite acceptable, whereas in other applications, such as periodontal bone defects repair, sinus lift, *etc*., the ability of the hardened cement to be replaced quickly by bone is crucial. Experimental results suggest that a number of parameters of calcium orthophosphate cements, such as the Ca/P ionic ratio, carbonate content, ionic substitution, crystallinity, *etc*. might affect the dissolution characteristics of the cements in slightly acidic solutions. This gives an opportunity to formulate cements, possessing different resorption rates, which is suited for different applications [117,118].

The discovery of calcium orthophosphate cements and concretes has already opened up new perspectives in synthesis of bioceramic scaffolds, possessing sufficient mechanical properties [249,250,274,336]. In the past, such scaffolds could only be manufactured by the sintering route at elevated temperatures. Therefore, until recently it was impossible to produce resorbable preset low-temperature hydrated 3D ceramics for various applications, *e.g.,* scaffolds and granules, from low-temperature calcium orthophosphate phases, such as ACP, DCPA, DCPD, OCP and CDHA. Now, using the appropriate techniques, open macroporous 3D scaffolds consisting of the aforementioned low-temperature phases (currently, excluding ACP and OCP) can be produced via a cementitious reaction [492,498,499,500,501], thus dramatically widening the application of these calcium orthophosphates as biomaterials and bioceramics. This type of materials could be very promising for tissue engineering applications. Among them, CDHA is of a special interest due to its chemical similarity to bone material and a large specific surface area.

To conclude this part, one should stress, that the most promising direction of the future developments of calcium orthophosphate cements and concretes is obviously seen in their functionalization by incorporation or impregnation of various hormones, growth factors, drugs, other bioorganic compounds, as well as incorporation of living cells and other tiny biological objects [502,503,504,505,506,507]. The initial attempts have already been performed but without a great success yet. For example, researchers have already found that unset calcium orthophosphate cements might have toxic effects when placed on cell monolayers, while the set cements are biocompartible for the same type of cells (MC3T3-E1 osteoblast-like cells were tested). A gel encapsulation in alginate beads was found to be a possible solution to protect living cells for seeding into calcium orthophosphate cement pastes [508]. *In vitro* cytotoxic effect of a calcium orthophosphate cement based on α-TCP was also observed [509]. In light of these results, the encapsulation approach [255] could potentially be used to seed a patient’s *ex vivo* expanded stem cells into a cement to create an osteoinductive bone graft substitute that could be used to treat that patient. However, this becomes more related to tissue engineering and biology, rather than to chemistry and material science.

Finally, besides the aforementioned chemical, material and biomedical improvements of calcium orthophosphate cements and concretes, one should not forget on a better design of both the mixing equipment and delivery (injection) techniques. As an example, the interested readers are referred to a new cannula to ease cement injection during vertebroplasty [510]; however, this subject is beyond the scope of current review.

## 10. Conclusions

Thus, among the diverse range of bone replacing biomaterials, calcium orthophosphate cements and concretes undoubtedly represent a distinct group because they are relatively simple materials formed by combining a calcium orthophosphate mixture with an aqueous solution. However, they symbolize an important breakthrough in the field of bone repair biomaterials, since they offer the possibility of obtaining thermally unstable calcium orthophosphates in a monolithic form at room or body temperature by means of a cementation reaction. This particular fabrication technique implies that the cements are moldable and therefore can adapt easily to the bone cavity providing a good fixation and the optimum tissue-biomaterial contact, necessary for stimulating bone ingrowth into them and their subsequent osteotransduction [25].

Unfortunately, the perfect grafting material does not yet exist. Calcium orthophosphate cements and concretes are not an exception to this statement. While possessing excellent biological properties (osteoconduction and, occasionally, osteoinduction), adequate setting time, excellent moldability and the capability to deliver different bone-enhancing proteins/antibiotics at a local level, unfortunately, the material lacks adequate mechanical properties for applications other than non-loaded surgical sites (see Table 4 for other details). Nevertheless, even in its present state calcium orthophosphate cements appear to be suitable for a number of applications. They can be injected into osteoporotic bone to reinforce it or can be used to make granules and blocks out of low-temperature calcium orthophosphates. Several types of calcium orthophosphate cements are now on the market, while scaffolds made of low-temperature calcium orthophosphates are being tested. The use of slightly different chemical compositions and various dopants affects both the setting time and tensile strength that enables further improvements. In addition, new trials are being conducted with reinforced formulations and concretes, which represent additional attempts to improve the existing products.

It is anticipated that the use of calcium orthophosphate cements will enable a faster and more aggressive rehabilitation, as the strength of the cement makes it possible to allow full weight-bearing earlier than when bone graft is used. Although, preliminary clinical trials have already confirmed the great potential of this novel therapeutic product, calcium orthophosphate cements need to be improved further; in particular, their bioresorption needs to be accelerated as well as their injectability and mechanical properties need to get better. Besides, extra clinical studies are required to define the most appropriate indications and limitations of calcium orthophosphate cements for fracture repair.

In the author’s humble opinion, mentioning Prof. James M. Anderson’s opinion on the history of biomaterials field would be the best way to conclude this review. According to Prof. Anderson, within 1950 – 1975 the researchers studied bioMATERIALS, within 1975 – 2000 they studied BIOMATERIALS and since 2000 the time for BIOmaterials has been coming [511]. Here, the capital letters emphasis the major direction of the research efforts in the complex subject of biomaterials. As the history of calcium orthophosphate cements started only in 1983, the aforementioned periods were shifted along the time scale. Certainly, the bioMATERIALS-epoch for calcium orthophosphate cements is almost over (every possible combination of the cement formulation has been already tested), while the BIOmaterials-era (where cells are the key factor) either has not started yet or is just at the very beginning. Most likely, current state-of-the-art of calcium orthophosphate cements and concretes corresponds to BIOMATERIALS-phase with an approximately equal contribution of biological and materials directions. Therefore, still there is much room for versatile ideas and approaches.
